# Chagas disease vector control and Taylor's law

**DOI:** 10.1371/journal.pntd.0006092

**Published:** 2017-11-30

**Authors:** Joel E. Cohen, Lucía I. Rodríguez-Planes, María S. Gaspe, María C. Cecere, Marta V. Cardinal, Ricardo E. Gürtler

**Affiliations:** 1 Laboratory of Populations, Rockefeller University, New York, NY, United States of America; 2 Earth Institute and Department of Statistics, Columbia University, New York, NY, United States of America; 3 Department of Statistics, University of Chicago, Chicago, IL, United States of America; 4 Universidad de Buenos Aires. Facultad de Ciencias Exactas y Naturales, Laboratory of Eco-Epidemiology, Ciudad Universitaria, Buenos Aires, Argentina; 5 Consejo Nacional de Investigaciones Científicas y Técnicas-Universidad de Buenos Aires. Instituto de Ecología, Genética y Evolución de Buenos Aires (IEGEBA), Ciudad Universitaria, Buenos Aires, Argentina; McGill University, CANADA

## Abstract

**Background:**

Large spatial and temporal fluctuations in the population density of living organisms have profound consequences for biodiversity conservation, food production, pest control and disease control, especially vector-borne disease control. Chagas disease vector control based on insecticide spraying could benefit from improved concepts and methods to deal with spatial variations in vector population density.

**Methodology/Principal findings:**

We show that Taylor's law (TL) of fluctuation scaling describes accurately the mean and variance over space of relative abundance, by habitat, of four insect vectors of Chagas disease (*Triatoma infestans*, *Triatoma guasayana*, *Triatoma garciabesi* and *Triatoma sordida*) in 33,908 searches of people's dwellings and associated habitats in 79 field surveys in four districts in the Argentine Chaco region, before and after insecticide spraying. As TL predicts, the logarithm of the sample variance of bug relative abundance closely approximates a linear function of the logarithm of the sample mean of abundance in different habitats. Slopes of TL indicate spatial aggregation or variation in habitat suitability. Predictions of new mathematical models of the effect of vector control measures on TL agree overall with field data before and after community-wide spraying of insecticide.

**Conclusions/Significance:**

A spatial Taylor's law identifies key habitats with high average infestation and spatially highly variable infestation, providing a new instrument for the control and elimination of the vectors of a major human disease.

## Author summary

Chagas disease, or American trypanosomiasis, is mainly transmitted to humans by insects that dwell in human habitations and structures closely associated with human habitations, such as kitchen out-buildings, chicken coops, goat corrals, and grain storage bins. Widespread in the Americas, the disease causes chronic illness and often eventual death. No vaccines exist. Available drugs may cause undesirable adverse effects and do not prevent re-infection. Efforts at suppressing the disease have been directed at eliminating the principal insect vector species from human dwelling compounds. Effective insecticide spraying requires finding out where the insects are. Both the average and the variance of the relative number of insect vectors of each species in each habitat are relevant to control efforts. We demonstrate here that the spatial distribution of the insect vectors of Chagas disease obeys a previously unrecognized pattern, known in ecology as Taylor's law (TL): in different habitats, the variance of vector relative numbers is approximately a power function of the mean of vector relative numbers. TL identifies key habitats with high average infestation and highly variable infestation, providing a new instrument for the control and elimination of the vectors of a major human disease.

## Introduction

### Chagas disease

Vector-borne pathogens contribute to 17% of the global human disease burden [1]. Chagas disease or American trypanosomiasis, one of the World Health Organization's "neglected tropical diseases," is caused by the protozoan *Trypanosoma cruzi*. It is transmitted mainly by diverse triatomine bug species associated with selected wild, peridomestic and domestic habitats in the Americas. The major vectors of human Chagas disease thrive in human dwellings and peridomestic structures housing domestic animals. (Peri)domestic populations of the major vector *Triatoma infestans* differ widely depending on the specific local habitat and host species [2]. Here "(peri)domestic" refers to structures that are domestic or peridomestic.

We show that Taylor's law (TL), which we describe below, describes well the average and variance of habitat-specific relative population sizes of *T*. *infestans* and three other vector species of *T*. *cruzi* in all (peri)domestic habitats. The data result from 33,908 habitat searches for triatomine bugs in four areas of Argentina from 1993 to 2010 before and after the large disturbance caused by community-wide insecticide spraying directed to suppress (peri)domestic infestations with *T*. *infestans*. One area, well described by TL, had moderate insecticide resistance that caused vector control failures. We determine the effect of insecticide spraying or the history of chemical control interventions on the values of the parameters of TL and describe some implications of TL for vector control and surveillance. The present paper may be the first to demonstrate the connection of TL with any aspect of Chagas disease, and in particular with the population densities of the insect vectors of the disease.

### Taylor's law

Large spatial and temporal fluctuations in the population density of living organisms have profound consequences for biodiversity conservation, food production, pest control and disease control, especially vector-borne disease control. In empirical studies of insects and many other species, the sample variance *v* and the sample mean *m* of population counts or other measures of population density or abundance approximate well a linear relationship on log-log coordinates, log_10_ *v* ≈ *a* + *b* * log_10_ *m* [3], which is mathematically equivalent to the power law *v* ≈ 10^*a*^*m*^*b*^. From a mathematical point of view, the slope *b* of TL is the proportional (or percentage) rate of increase in the variance for a given infinitesimal proportional (or percentage) increase in the mean. The slope *b* has been interpreted as an index of spatial aggregation because purely random (Poisson) distributions of individuals have a variance equal to the mean and therefore would be expected to generate TL with *b* = 1, while some distributions in which the variance grows faster than in proportion to the mean would be expected to generate TL with *b* > 1. Variations in habitat suitability and other ecological mechanisms could also generate TL with *b* > 1. (See *Future research* in the Discussion.)

Although Taylor was not the first to publish empirical examples of the above linear relationship, he made it widely known [4,5], and it is usually called Taylor's law (TL) among ecologists, or fluctuation scaling or large-number scaling among physicists [6]. More than 1000 papers have been published on TL and its applications to hundreds of species and many fields besides ecology [6], including weekly cases of measles in 366 communities in England and Wales pre- and post-vaccination [7], the aggregation of parasite individuals within host individuals (not including any parasites, vectors, or hosts related to the transmission of Chagas disease) [8,9], human population densities [10], crop yields [11], prime numbers [12] and tornado outbreaks [13]. TL can be generated by many different models (e.g., [6,7,14–16]).

TL has important applications in the management of agricultural pests and fisheries. When TL is valid, TL can be used to design more efficient sampling schemes to estimate pest density and decide whether to spray pesticides or release natural enemies in a timely fashion [17, 18]. TL provides a stopping rule for fixed precision sampling of fisheries, permitting reduced sampling effort [19].

The uses of TL to identify unusual variability in crop yields [11] and plan more efficient control measures by recognizing the heteroskedasticity of population densities at different mean densities are potentially valid for controlling the insect vectors of major human infectious diseases, including malaria, dengue, Chagas disease, sleeping sickness and the leishmaniases. However, literature searches in Pubmed and Google Scholar (October 16, 2017) using "Taylor’s law" (or "Taylor’s power law") combined with "malaria mosquito (or *Anopheles*)", or "dengue mosquito (or *Aedes aegypti*)", or "tsetse fly (or *Glossina* fly)", or "Chagas vector (or *Triatoma*)", or "Leishmaniasis sandfly (or *Lutzomyia* or *Phlebotomus*)", identified no paper on Chagas disease vectors and TL and only a few papers which mainly used TL for sample size determination of malaria and dengue mosquitoes [20–29].

Two widely tested forms of TL are a temporal TL and a spatial TL. In a temporal TL, *n* populations labeled *i* = 1, …, *n* are followed over time, and the sample mean size (averaged over time) *m*_*i*_ of population *i* and the sample variance of population size (over time) *v*_*i*_ of population *i* are calculated separately for each population *i*. Each population is represented by one dot associated with population *i* on a plot of log_10_ *v*_*i*_ (vertical axis) as a function of log_10_ *m*_*i*_ (horizontal axis). If the dots fall approximately along a straight line, the data support a temporal TL.

In a spatial TL, which we pursue here, different populations of a species are grouped into different categories. In this article, each category will be a different habitat in which Chagas vectors may be found, such as a chicken coop or a goat corral. Habitats are labeled *h* = 1, …, *H*, where *H* is the number of different habitats. The mean *m*_*h*_ and the variance *v*_*h*_ of population sizes over all sites of habitat *h* (e.g., over all chicken coops in a community) are calculated and log_10_ *v*_*h*_ is plotted as a function of log_10_ *m*_*h*_, with one data point for each habitat *h*. If the *H* dots fall approximately along a straight line, the data support a spatial TL.

### Purposes of this article

We adopt some conventions of language. We use "areas" to refer collectively to the four geographical locations, Amamá, Olta, Figueroa, and Pampa del Indio, where studies and control efforts were conducted. We use "habitat" for a category of individual places that were surveyed for bugs. For example, chicken coops are one habitat, goat corrals are another habitat, and cow corrals are a third habitat. We use "site" for a particular exemplar of a habitat, such as a particular chicken coop, or a particular goat corral. A "house compound" consists of a domicile for people and near-by buildings for human use and corrals for animals. Each such domicile and building is one site. As indicated above, "(peri)domestic" habitats include all such structures.

Here we demonstrate that TL describes the spatial distribution (in different sites of a habitat) of four of the vector species of Chagas disease. When the means and variances of the number of each species of vector are computed over sites separately for each habitat in a community of house compounds, TL is confirmed with high accuracy and consistency over time and under diverse control procedures. The slope *b* of TL does not deviate significantly from the range 1 < *b* < 2. We develop simple mathematical models to help interpret and extend this primary empirical finding. We suggest some practical consequences and potential uses of TL in Chagas disease vector control. The full implications of TL for Chagas disease vector control remain to be worked out in future research and practice and are not the primary objective of this paper. Finally, we suggest some future research.

## Methods and materials

### Field data

**Fig 1:**
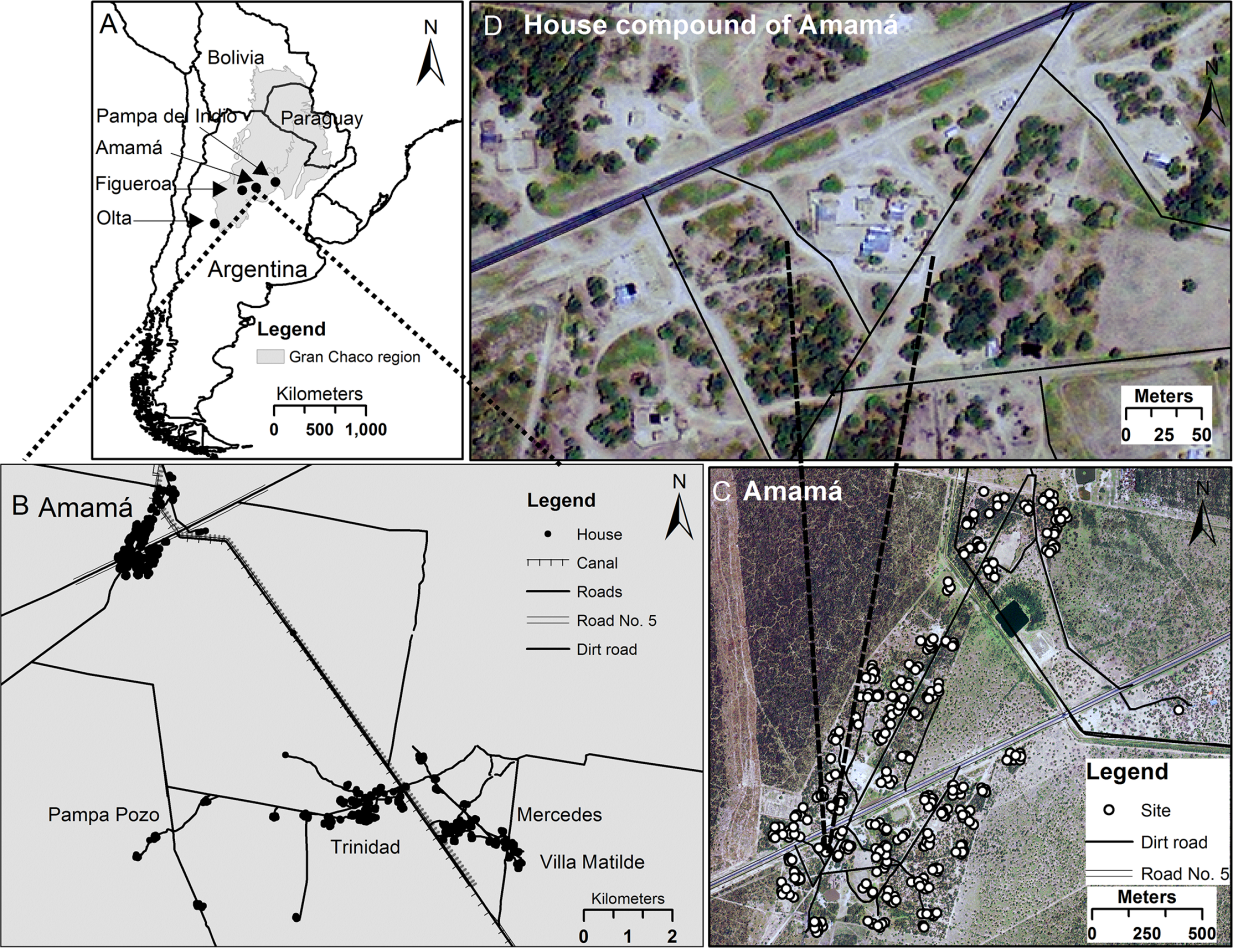
Map of the study areas, illustrated by the example of the Amamá study area, to be read counterclockwise from the upper left corner. (A) Gran Chaco region of northwest Argentina and neighboring countries, including the four study areas (Amamá, Olta, Figueroa, and Pampa del Indio). (B) Amamá study area core (Amamá village, Trinidad, Mercedes, Villa Matilde and Pampa Pozo). (C) Amamá village, showing individual sites (open circles). (D) One house compound in Amamá village, showing individual buildings.

The data come from four large research projects in the Argentine Chaco region where Chagas disease was endemic. These projects aimed primarily to control the major vector *Triatoma infestans*, but also included observations of other local triatomines not considered as the main control targets. The surveys were conducted in well-defined rural areas of Olta (municipalities of General Belgrano and Chamical, of the province of La Rioja, western Argentina), Figueroa and Amamá (Figueroa and Moreno departments, respectively, of the province of Santiago del Estero, northwestern Argentina), and Pampa del Indio (General San Martín department, of the province of Chaco, northeastern Argentina). The studies were organized spatially in a hierarchy with five levels: Argentine Chaco region; four study areas within the region; villages within each area; house compounds (defined above under "Purposes") within each village; and sites within each house compound. The details of each area are described extensively in Detailed Methods in S1 Text. Fig 1(A) maps the areas of these studies and Fig 1B, 1C and 1D illustrates the hierarchy of villages, house compounds, and sites. Table 1 summarizes the quantities of the data collected.

**Table 1:** Summary of the main activities and data collected in each of the study areas, Amamá (core and periphery), Olta, Figueroa, Pampa del Indio, Argentina.

	Study location				
Variable	Amamá core	Amamá periphery	Olta	Figueroa	Pampa del Indio
Date of first survey	October 1993	May 2003	April 1999	October 2003	October 2007
Date(s) of community-wide insecticide spraying	October 1985 (Amamá only); October 1992 and April 2004 (all villages)	April 2004	December 1999	November 2003	November 2007
Date of last vector survey	October 2002	May 2003	December 2000	March 2005	October 2010
Number of surveys	13	1	2	4	8
Number of villages	5	35	76	4	13
Number of house compounds inspected for infestation (range)	93–131	186	325–362	126–209	319–329
Number of types of habitats	17	16	15	18	20
Number of sites inspected at the first/last survey	438/646	702	860/860	1246/1398	2200/2409
Number of *T*. *infestans* collected at the first/last survey	12/496	901	4648/1686	1274/692	2035/19
% of house compounds infested with *T*. *infestans* in domestic habitats at last survey	11.7	17.6	ND	23.7	0.3
% of house compounds infested with *T*. *infestans* in peridomestic habitats at last survey	31.3	50.7	90.1	27.2	0.6
% of sites infested with *T*. *infestans* at last survey	10.5	16.0	28.1	9.9	0.2
Number of habitats with mean bug abundance of *T*. *infestans* greater than zero	11	13	15*	9	15
*T*. *infestans* present	yes	yes	yes	yes	yes
*T*. *guasayana* present	yes	yes	yes	yes	no
*T*. *garciabesi* present	yes	yes	yes	yes	no
*T*. *sordida* present	no	no	no	no	yes
Triatomine count techniques	Timed-manual collections	Timed-manual collections	Timed-manual collections	Timed-manual collections	Timed-manual collections
Data sets analyzed	core vs periphery / core longitudinal	core vs periphery	pre vs post	pre vs post	pre vs post
Key references	[30,31]	[30,32]	[33]	[34]	[35,36]

* Eight habitats with adequate sample size included in the analysis

#### Data sets

S1 Table summarizes, for *T*. *infestans* only, the sample means and sample variances of relative bug population density, by study, by survey, and by habitat. S2 Table gives the raw data for all species. From these data, we extracted 83 sets of data from which we estimated a slope and intercept of TL (Results, Table 2), as described below. From these 83 regressions, we selected 79 with sufficient observations to support analysis of the variability of parameter estimates of individual species.

**Table 2:** Linear regression estimates of the parameters of Taylor's law (TL) log_10_ *v* = *a* + *b* log_10_ *m*, with *m* = sample mean, *v* = sample variance of the relative abundance of triatomines collected in domestic and peridomestic habitats from four field studies, separately for each vector species, intervention status, and survey (83 cases). The Note below the table defines column headings and explains entries where necessary.

Dataset	Triatoma species		Intervention	Minimum Log Mean	Maximum Log Mean	Range Log Mean	Intercept_a	SE_a	Slope_b	SE_b	Adj R^2^	df &	Test_a_0	Test_c_0	Swilk	Hetero- skedasticity	Information Matrix
Amamá comparative		T. infestans	Pulsed/sustained control pooled	-2.061	0.388	2.449	1.310	0.082	1.633	0.089	0.960	13	<0.001	ns	ns	ns	ns
Amamá comparative		T. infestans	Sustained control (core)	-2.000	1.000	3.000	1.070	0.105	1.610	0.112	0.954	9	<0.001	ns	ns	ns	ns
Amamá comparative		T. infestans	Pulsed control (periphery)	-1.881	0.713	2.594	1.080	0.087	1.576	0.106	0.948	11	<0.001	ns	ns	ns	ns
Amamá comparative		T. guasayana	Pulsed/sustained control pooled	-2.061	-0.952	1.109	0.589	0.250	1.312	0.157	0.896	7	0.05	ns	ns	0.03	0.02
Amamá comparative		T. guasayana	Sustained control (core)	-2.000	-0.671	1.329	0.799	0.261	1.440	0.173	0.907	5	0.022	ns	ns	ns	ns
Amamá comparative		T. guasayana	Pulsed control (periphery)	-1.881	-0.477	1.404	0.055	0.198	0.965	0.166	0.845	5	ns	ns	0.001	ns	ns
Amamá comparative		T. garciabesi	Pulsed/sustained control pooled	-1.959	-0.219	1.740	1.040	0.174	1.534	0.134	0.956	5	0.002	ns	ns	ns	ns
Amamá comparative		T. garciabesi	Sustained control (core)	-1.633	0.076	1.709	0.681	0.144	1.310	0.138	0.947	3	0.009	ns	0.068	ns	ns
Amamá comparative		T. garciabesi	Pulsed control (periphery)	-1.156	-0.418	0.738	0.435	0.437	1.065	0.522	0.442	3	ns	ns	ns	ns	ns
Amamá longitudinal		T. infestans	Core postintervention Oct93	-1.748	-0.602	1.146	0.320	0.450	1.155	0.369	0.687	3	ns	ns	<0.001	ns	ns
Amamá longitudinal		T. infestans	Core postintervention Nov94	-2.061	0.653	2.714	0.532	0.157	1.357	0.118	0.970	3	0.043	0.026	0.056	ns	ns
Amamá longitudinal		T. infestans	Core postintervention May95	-1.771	-1.130	0.641	0.687	0.363	1.422	0.248	0.914	2	ns	ns	ns	ns	ns
Amamá longitudinal		T. infestans	Core postintervention Nov95	-1.556	-0.026	1.530	0.938	0.109	1.487	0.116	0.970	4	0.001	0.051	ns	ns	ns
Amamá longitudinal		T. infestans	Core postintervention May96	-1.204	1.079	2.283	0.810	0.137	1.531	0.142	0.951	5	0.002	ns	ns	ns	ns
Amamá longitudinal		T. infestans	Core postintervention Nov96	-1.342	-0.073	1.270	0.852	0.163	1.571	0.199	0.911	5	0.003	ns	ns	ns	ns
Amamá longitudinal		T. infestans	Core postintervention May97	-0.929	-0.176	0.753	1.168	0.327	2.002	0.720	0.529	5	0.016	ns	ns	ns	ns
Amamá longitudinal		T. infestans	Core postintervention Nov97	-1.301	0.551	1.852	0.944	0.075	1.927	0.118	0.964	9	<0.001	ns	ns	ns	ns
Amamá longitudinal		T. infestans	Core postintervention May98	-1.869	-0.206	1.663	1.098	0.262	1.633	0.260	0.885	4	0.014	ns	ns	ns	ns
Amamá longitudinal		T. infestans	Core postintervention Dec98	-1.826	-0.044	1.782	1.235	0.110	1.628	0.106	0.971	6	<0.001	0.040	ns	ns	ns
Amamá longitudinal		T. infestans	Core postintervention May99	-2.130	-0.094	2.036	1.061	0.130	1.577	0.117	0.947	9	<0.001	ns	ns	ns	ns
Amamá longitudinal		T. infestans	Core postintervention Mar00	-2.134	0.709	2.842	0.962	0.092	1.565	0.101	0.952	11	<0.001	ns	ns	ns	ns
Amamá longitudinal		T. infestans	Core postintervention Oct02	-2.000	1.000	3.000	1.070	0.105	1.610	0.112	0.954	9	<0.001	ns	ns	ns	ns
Amamá longitudinal		T. guasayana	Core postintervention Oct93	-1.195	-0.778	0.417	-0.882		-0.133			0					
Amamá longitudinal		T. guasayana	Core postintervention Nov94	-2.061	-0.699	1.362	0.044	0.214	0.970	0.144	0.881	5	ns	ns	<0.001	ns	ns
Amamá longitudinal		T. guasayana	Core postintervention May95	-2.045	0.148	2.194	0.991	0.183	1.533	0.171	0.963	2	0.033	ns	ns	ns	ns
Amamá longitudinal		T. guasayana	Core postintervention Nov95	-1.613	0.301	1.914	0.874	0.108	1.512	0.084	0.991	2	0.015	ns	ns	ns	ns
Amamá longitudinal		T. guasayana	Core postintervention May96	-1.505	-0.075	1.430	0.623	0.201	1.447	0.187	0.868	8	0.015	ns	ns	ns	ns
Amamá longitudinal		T. guasayana	Core postintervention Nov96	-2.097	-0.354	1.743	0.516	0.208	1.301	0.141	0.944	4	0.068	0.007	ns	ns	ns
Amamá longitudinal		T. guasayana	Core postintervention May97	-1.255	-0.013	1.242	0.812	0.223	1.687	0.240	0.890	5	0.015	ns	ns	ns	ns
Amamá longitudinal		T. guasayana	Core postintervention Nov97	-2.029	-0.103	1.927	0.556	0.141	1.358	0.106	0.964	5	0.011	0.009	ns	ns	ns
Amamá longitudinal		T. guasayana	Core postintervention May98	-2.053	-0.125	1.928	0.703	0.105	1.351	0.077	0.984	4	0.003	ns	ns	0.036	ns
Amamá longitudinal		T. guasayana	Core postintervention Dec98	-2.083	-0.602	1.481	0.216	0.171	1.043	0.114	0.922	6	ns	0.048	<0.001	ns	ns
Amamá longitudinal		T. guasayana	Core postintervention May99	-1.562	-0.947	0.615	1.893	0.438	2.270	0.330	0.959	1	ns	-	0.025	ns	ns
Amamá longitudinal		T. guasayana	Core postintervention Mar00	-1.531	-0.352	1.179	0.594	0.197	1.282	0.170	0.933	3	0.057	ns	ns	ns	ns
Amamá longitudinal		T. guasayana	Core postintervention Oct02	-2.000	-0.671	1.329	0.799	0.261	1.440	0.173	0.907	6	0.022	ns	ns	ns	ns
Amamá longitudinal		T. garciabesi	Core postintervention Oct93	-1.778	-0.301	1.477	0.496	0.175	1.297	0.149	0.949	3	0.065	ns	0.045	ns	0.049
Amamá longitudinal		T. garciabesi	Core postintervention Nov94	-1.342	0.477	1.820	0.708	0.153	1.418	0.239	0.791	8	0.002	ns	ns	ns	ns
Amamá longitudinal		T. garciabesi	Core postintervention May95	-0.845	-0.125	0.720	1.021	0.123	1.680	0.235	0.926	3	0.004	ns	ns	ns	ns
Amamá longitudinal		T. garciabesi	Core postintervention Nov95	-2.049	-0.106	1.943	0.529	0.116	1.303	0.104	0.940	9	0.001	ns	0.056	ns	ns
Amamá longitudinal		T. garciabesi	Core postintervention May96	-1.591	0.374	1.965	0.871	0.093	1.596	0.131	0.937	9	<0.001	ns	ns	ns	ns
Amamá longitudinal		T. garciabesi	Core postintervention Nov96	-1.663	0.240	1.902	0.819	0.110	1.436	0.121	0.952	6	<0.001	ns	ns	ns	ns
Amamá longitudinal		T. garciabesi	Core postintervention May97	-1.362	0.357	1.718	1.008	0.089	1.770	0.085	0.984	6	<0.001	ns	ns	ns	ns
Amamá longitudinal		T. garciabesi	Core postintervention Nov97	-1.455	0.407	1.862	0.843	0.126	1.480	0.151	0.941	5	0.001	ns	ns	ns	ns
Amamá longitudinal		T. garciabesi	Core postintervention May98	-1.398	0.097	1.495	0.664	0.136	1.454	0.130	0.954	5	0.005	ns	ns	ns	ns
Amamá longitudinal		T. garciabesi	Core postintervention Dec98	-1.415	-0.301	1.114	1.005	0.226	1.724	0.203	0.934	4	0.011	ns	ns	ns	ns
Amamá longitudinal		T. garciabesi	Core postintervention May99	-1.792	0.398	2.190	1.112	0.110	1.614	0.086	0.986	4	0.001	ns	ns	ns	ns
Amamá longitudinal		T. garciabesi	Core postintervention Mar00	-1.519	-0.109	1.409	0.685	0.115	1.394	0.112	0.951	7	0.001	ns	ns	ns	ns
Amamá longitudinal		T. garciabesi	Core postintervention Oct02	-1.633	0.076	1.709	0.681	0.144	1.310	0.138	0.947	4	0.009	ns	0.068	ns	ns
Olta		T. infestans	Preintervention	-0.004	0.968	0.972	1.100	0.105	1.233	0.150	0.905	6	<0.001	ns	0.031	ns	ns
Olta		T. infestans	Postintervention	-0.390	0.481	0.871	1.095	0.118	1.767	0.347	0.781	6	<0.001	0.032	0.090	ns	ns
Olta		T. guasayana	Preintervention	-1.498	-0.092	1.406	0.765	0.157	1.428	0.167	0.912	6	0.003	ns	0.044	ns	ns
Olta		T. guasayana	Postintervention	-2.170	-0.308	1.862	0.638	0.144	1.343	0.096	0.964	6	0.004	ns	0.074	ns	ns
Olta		T. garciabesi	Preintervention	-1.431	-0.561	0.870	1.007	0.199	1.558	0.190	0.930	4	0.007	ns	ns	0.031	ns
Olta		T. garciabesi	Postintervention	-1.699	-0.130	1.569	0.745	0.128	1.435	0.100	0.981	3	0.010	ns	0.041	ns	ns
Olta		Three sp. Combined	Preintervention	0.156	0.986	0.830	0.995	0.122	1.332	0.168	0.898	6	<0.001	ns	ns	ns	ns
Olta		Three sp. Combined	Postintervention	-0.357	0.551	0.908	1.037	0.083	1.646	0.240	0.868	6	<0.001	ns	ns	0.060	0.036
Figueroa		T. infestans	Preintervention Oct03	-1.283	0.513	1.795	1.129	0.072	1.401	0.146	0.919	7	<0.001	ns	ns	ns	ns
Figueroa		T. infestans	Postintervention Mar04	-0.723	0.410	1.133	1.065	0.108	2.092	0.225	0.934	5	<0.001	ns	ns	ns	ns
Figueroa		T. infestans	Postintervention Oct04	-1.447	0.222	1.669	1.294	0.127	1.821	0.152	0.941	8	<0.001	0.021	ns	ns	ns
Figueroa		T. infestans	Postintervention Mar05	-1.678	0.066	1.744	1.239	0.083	1.562	0.133	0.945	7	<0.001	0.006	0.063	ns	ns
Figueroa		T. guasayana	Preintervention Oct03	-1.823	-1.230	0.593	0.887	0.676	1.468	0.480	0.677	3	ns	ns	ns	ns	ns
Figueroa		T. guasayana	Postintervention Oct04	-2.097	-1.301	0.796	-0.043	0.017	0.978	0.010	1.000	2	ns	ns	ns	ns	ns
Figueroa		T. guasayana	Postintervention Mar05	-2.088	-0.894	1.194	0.850	0.382	1.351	0.255	0.795	6	0.068	ns	ns	ns	ns
Figueroa		T. garciabesi	Preintervention Oct03	-2.274	-1.623	0.651	0.000	0.000	1.000	0.000	1.000	4	ns	ns	ns	ns	
Figueroa		T. garciabesi	Postintervention Mar05	-2.143	-0.736	1.407	1.020	0.430	1.441	0.301	0.814	4	0.077	ns	ns	ns	ns
Pampa del Indio		T. infestans	Preintervention Oct07	-1.871	0.395	2.267	1.384	0.105	1.601	0.112	0.944	11	<0.001	ns	ns	ns	ns
Pampa del Indio		T. infestans	Postintervention Apr08	-1.593	-0.396	1.197	1.515	0.276	1.738	0.297	0.769	9	<0.001	ns	ns	ns	ns
Pampa del Indio		T. infestans	Postintervention Oct08	-1.463	-0.136	1.328	1.429	0.159	1.438	0.203	0.860	7	<0.001	ns	ns	ns	ns
Pampa del Indio		T. infestans	Postintervention Dec08	-1.872	0.121	1.994	1.224	0.222	1.356	0.181	0.888	6	0.002	ns	ns	ns	ns
Pampa del Indio		T. infestans	Postintervention May09	-1.851	-0.480	1.371	1.902	0.198	2.007	0.175	0.936	8	<0.001	ns	ns	ns	ns
Pampa del Indio		T. infestans	Postintervention Oct09	-2.196	-1.632	0.564	2.460	1.245	2.145	0.665	0.758	2	0.187	ns	ns	ns	ns
Pampa del Indio		T. infestans	Postintervention Apr10	-1.600	-0.467	1.133	1.402	0.251	1.387	0.205	0.918	3	0.011	ns	ns	ns	ns
Pampa del Indio		T. infestans	Postintervention Oct10	-2.601	-0.934	1.666	1.822	0.226	1.752	0.112	0.988	2	0.015	ns	ns	ns	ns
Pampa del Indio		T. infestans	All sites, given dates	-2.291	-0.034	2.257	1.592	0.092	1.299	0.063	0.984	6	<0.001	ns	ns	0.013	0.066
Pampa del Indio		T. sordida	Preintervention Oct07	-2.091	-0.010	2.081	1.249	0.135	1.671	0.115	0.942	12	<0.001	ns	ns	0.056	ns
Pampa del Indio		T. sordida	Postintervention Apr08	-2.463	-0.119	2.344	1.060	0.200	1.508	0.141	0.912	10	<0.001	ns	ns	ns	ns
Pampa del Indio		T. sordida	Postintervention Oct08	-2.640	-0.820	1.819	0.778	0.165	1.316	0.095	0.951	9	0.001	ns	ns	ns	ns
Pampa del Indio		T. sordida	Postintervention Dec08	-2.328	-0.009	2.319	1.542	0.165	1.748	0.129	0.958	7	<0.001	ns	ns	ns	ns
Pampa del Indio		T. sordida	Postintervention May09	-2.188	0.304	2.492	1.319	0.152	1.660	0.132	0.941	9	<0.001	ns	ns	ns	ns
Pampa del Indio		T. sordida	Postintervention Oct09	-2.179	-0.128	2.051	1.336	0.146	1.626	0.122	0.942	10	<0.001	colinear	ns	ns	ns
Pampa del Indio		T. sordida	Postintervention Apr10	-2.140	0.543	2.683	1.211	0.099	1.543	0.087	0.963	11	<0.001	colinear	ns	ns	ns
Pampa del Indio		T. sordida	Postintervention Oct10	-2.292	-0.091	2.201	1.091	0.145	1.449	0.106	0.954	8	<0.001	colinear	ns	ns	ns
Pampa del Indio		T. sordida	All sites, given dates	-1.593	-0.505	1.088	1.979	0.275	1.810	0.267	0.865	6	<0.001	ns	ns	0.090	ns

Dataset: study area. For Amamá, "comparative" compares "core" villages, which had sustained vector control, with "periphery," outlying villages with pulsed vector control. "Longitudinal" presents successive surveys of the core. Triatoma species: "T." = "Triatoma." Intervention: date of survey and whether the survey preceded or followed community-wide spraying. Minimum Log Mean: Minimum over all habitats *h* with at least one infestation of the log_10_ of the sample mean *m*_*h*_ bug density in habitat *h*. Maximum Log Mean: Maximum over all habitats *h* of the log_10_ of the sample mean *m*_*h*_ bug density. Range Log Mean: Maximum Log Mean minus Minimum Log Mean, to indicate the range of the abscissa in the scatterplot of TL. Intercept_a: least-squares estimate of the intercept *a* of the log-log form of Taylor's law (TL) log_10_ *v* ≈ *a* + *b* × log_10_ *m*. SE_a: standard error of the estimate of *a*. Slope_b: least-squares estimate of the slope *b* of TL. SE_b: standard error of the estimate of *b*. Adj R^2^: adjusted *R*^2^ (adjusted for the number of fitted parameters). df: error (residual) degrees of freedom (df) equals number of observations minus the number of fitted parameters. Test_a_0: p-value of the test of the null hypothesis that the intercept *a* equals 0. Test_c_0: p-value of the test of the null hypothesis that the coefficient *c* of the quadratic term equals 0. A low p-value rejects the adequacy of TL because the relationship of log_10_ *v* to log_10_ *m* has statistically significant curvature. "ns" = not significant at the 5% level. Colinear: c could not be estimated due to significant collinearity between the linear and the quadratic terms. Swilk: Shapiro-Wilk test of the normality of residuals from the linear regression of TL. A low p-value rejects the assumption of normally distributed residuals. Heteroskedasticity: p-value of the test of the null hypothesis that the residuals from the linear regression are homoskedastic, i.e., all have the same variance. Information Matrix: p-value of the test of the null hypothesis for heteroskedasticity, skewness, and kurtosis.

### Statistical analysis

#### Fitting and testing Taylor's law: Summary

We fitted TL and tested its adequacy as a description of the data in three steps. First, for every habitat *h* in an area (e.g., domiciles, kitchens, chicken coops, pig corrals), we computed the sample mean *m*_*h*_ and the sample variance *v*_*h*_ of the number of bugs in each site of that habitat per standardized search effort (as described in Detailed Methods) and executed ordinary least-squares linear regression of log_10_ *v*_*h*_ on log_10_ *m*_*h*_, *h* = 1, …, *H*, across all habitats in the area, separately for each triatomine species, survey, and area. Second, we tested for curvature in the relation of log_10_ *v*_*h*_ to log_10_ *m*_*h*_ by fitting a quadratic regression log_10_ *v*_*h*_ = *a* + b* log_10_ *m*_*h*_ + c*(log_10_ *m*_*h*_)^2^ by least squares. If the confidence interval of the coefficient *c* did not include 0, the data rejected TL because there was statistically significant evidence of curvature. In most cases, there was no statistically significant evidence of curvature, and we examined the residuals of the linear regression models for heteroskedasticity, normality, skewness and kurtosis. Third, when the analyses in steps 1 and 2 did not reject TL as a description of the data, we used analysis of covariance (ANCOVA) to test for differences in the parameters of TL fitted to different subsets of the data. The following subsection gives the details of each step and the standard software used.

#### Fitting and testing Taylor's law: Details

The fit of the data to TL was tested by ordinary least-squares linear regression of log_10_ *v*_*h*_ on log_10_ *m*_*h*_, *h* = 1, …, *H*, separately for each triatomine species, survey, and area. Regression analyses were performed with Stata 14.2 [37]. The mean and variance of relative bug abundance for a given triatomine species, habitat, survey, and area were calculated over all identified sites that had been examined for infestation at a given point in time. Analyses included all habitats with mean abundance greater than 0, possibly including some individual sites with abundance equal to 0. Taylor et al. ([38], p. 721) suggested that at least 15 observations (here sites) should be available to calculate each mean and variance (here, for a given habitat) and that the linear regression (here, for a given species, survey and area) should include at least 5 paired data of *v*_*h*_ and *m*_*h*_. The data used here nearly always complied with these suggestions. For example, the Amamá longitudinal data on *T*. *guasayana* from the core postintervention surveys of October 1993 and May 1999 are included among the 83 regression estimates in Results, Table 2, but are omitted from further analyses because only two or three habitats had sufficient information to estimate means and variances, giving zero or one df for estimation of TL.

For Amamá core and periphery, we included a regression in Table 2 for the pooled data from both zones for each of the triatomine species. To pool the data, we collapsed all the raw data into one file that did not distinguish between zones. For Amamá, each habitat appeared exactly once, even if some habitats may have appeared in one zone and not in the other.

For Olta before and after intervention, Table 2 includes a regression for the pooled data from all of the triatomine species combined. To pool the data, we collapsed all the raw data into one file that did not distinguish between species. However, we excluded these two regressions from further analysis because the regressions were not species-specific. Our further analyses rest on the remaining 79 = 83–2–2 regressions used to estimate and test TL.

In a second step, we tested for curvature in the relation of log_10_ *v*_*h*_ to log_10_ *m*_*h*_ by fitting a quadratic generalization of TL originally suggested by Taylor et al. [39, p. 388, their equation (14)] and widely used since: log_10_ *v*_*h*_ = *a* + *b* log_10_ *m*_*h*_ + *c*(log_10_ *m*_*h*_)^2^. If the confidence interval of the coefficient *c* did not include 0, the data rejected TL because there was statistically significant curvature.

Residuals of the linear regression models were tested for normality, skewness and kurtosis using the commands swilk, estat hottest and estat imtest. The program swilk carries out the Shapiro-Wilk test; estat hottest performs three versions of the Breusch-Pagan [40] and Cook-Weisberg [41] test for heteroskedasticity vs homoskedasticity, and estat imtest performs an information matrix test for the regression model and an orthogonal decomposition into tests for heteroskedasticity, skewness and kurtosis [42]. Each residual of the TL linear regression measured the stability of population abundance in the corresponding habitat following [11]. In most cases of fitting the quadratic generalization of TL, the quadratic term was not significant, so these tests of the normality, skewness and kurtosis of the residuals were not performed for the quadratic regressions.

In a third step, when the analyses in steps 1 and 2 did not reject TL, we used analysis of covariance (ANCOVA, implemented in the anova command) to test for differences in the parameters of TL fitted to different subsets of the data. For example, when TL described acceptably the relationship between log sample mean and log sample variance of relative population density among different habitats of two or more species separately, we used ANCOVA to examine whether one or both of the parameters (slope and intercept) of the species-specific TLs differed between species. This ANCOVA treated "species" as a categorical variable and asked whether "species" or the interaction term "species × log sample mean" significantly influenced log sample variance. If "species" influenced log sample variance but not the interaction term, then the intercept of TL differed between species. If the interaction term influenced log sample variance, then "species" affected the slope. If both "species" and the interaction term influenced log sample variance, then both the intercept and the slope of TL depended on the species. We also used ANCOVA to test whether the parameters of TL for a given species differed before and after spraying of insecticides, or according to the history of control measures (sustained versus pulsed).

To compare estimates of slope under two conditions, we used Welch's t-test for two quantities with unequal variances [43]. These calculations used Matlab Version 9.2.0.556344 [44].

### Theory

Suppose that Taylor's law (TL) describes well the relation between the mean and the variance of relative population density of a single vector species in the habitats of a study area before the house compounds (including all (peri)domestic structures) are sprayed with insecticides to kill the vectors. What would we expect to be the effect of spraying? Specifically, would we expect TL to hold after spraying? If so, what if any connection should we expect between the intercept and slope of TL before and after spraying? Here we propose two simple models to answer these questions. In the Results, we will compare some of the predictions of these models with observations. It is not necessary to follow the mathematical details to understand the models' predictions or the empirical results. S1 Text gives mathematical proofs.

Both models use the same general notation. Suppose there are *H* > 2 habitats, such as chicken coop; open shed; oven; piled materials; cow corral; latrine/bathroom; etc. These habitats are labeled *h* = 1, 2, …, *H*. Let *B*(*h*) be a random variable representing the number of vectors of a single species (not all *Triatoma* species combined) in the various sites of habitat *h* in the study area *Before* spraying, and let *A*(*h*) be a random variable representing the number of vectors in the various sites of habitat *h* in the study area *After* spraying. Because of the gap in time between the survey before spraying and the survey after spraying, the set of sites of a given habitat before spraying may differ from the set of sites of that habitat after spraying.

The population mean (or expectation) of *B*(*h*) will be written *E*(*B*(*h*)) and the population variance, *Var*(*B*(*h*)); likewise, the population mean *E*(*A*(*h*)) and population variance *Var*(*A*(*h*)) of *A*(*h*). We assume the population mean and population variance exist and are positive. For the vectors before spraying, the log-log form of TL using the population mean *E*(*B*(*h*)) and the population variance *Var*(*B*(*h*)) instead of the corresponding sample mean *m* and sample variance *v* is log_10_ *Var*(*B*(*h*)) = *a* + *b* log_10_ *E*(*B*(*h*)). This linear form is mathematically equivalent to the power-law form of TL, namely, *Var*(*B*(*h*)) = *C*[*E*(*B*(*h*))]^*b*^, with *C* = 10^*a*^. The value of the slope *b* in the log-log form of TL is identical to the value of the exponent *b* in the power-law form, hence we use the same notation *b* and we refer to *b* interchangeably as the slope or the exponent. The value of the intercept *a* in the log-log form is related to the value of the coefficient *C* in the power-law form by 10^*a*^ = *C* or log_10_ *C* = *a*, hence we use different words and symbols (intercept *a* versus coefficient *C*).

We assume that spraying reduces the relative population density of the vector, and does not increase it.

#### Model 1: Constant survival proportion after spraying

Suppose that a fraction *s*, where 0 < *s* < 1, of vectors survive spraying. The letter *s* was chosen as a mnemonic for *Survive Spraying*. Model 1 assumes that this fraction *s* is the same for every site of a given habitat (e.g., for every chicken coop in the study area) and for all habitats (e.g., all chicken coops, cow corrals, etc.).

Then for every habitat *h* = 1, 2, …, *H*, we have *A*(*h*) = *sB*(*h*). Hence *E*(*A*(*h*)) = *E*(*sB*(*h*)) = *sE*(*B*(*h*)). Thus *E*(*B*(*h*)) = *s*^-1^*E*(*A*(*h*)). Also *Var*(*A*(*h*)) = *Var*(*sB*(*h*)) = *s*^2^*Var*(*B*(*h*)). Now if the relative population density of vectors satisfies TL before spraying, namely, *Var*(*B*(*h*)) = *C*[*E*(*B*(*h*))]^*b*^, then substituting TL into the prior equation and using *E*(*B*(*h*)) = *s*^-1^*E*(*A*(*h*)) give *Var*(*A*(*h*)) = *s*^2^*C*[*E*(*B*(*h*))]^*b*^ = *s*^2^ *C*[*s*^-1^*E*(*A*(*h*))]^*b*^ = *s*^2-*b*^*C*[*E*(*A*(*h*))]^*b*^. Thus *Var*(*A*(*h*)) = *s*^2-*b*^*C*[*E*(*A*(*h*))]^*b*^. TL holds exactly for the relative population density of vectors after spraying with the same exponent *b* but the coefficient *C* before spraying changes to *s*^2-*b*^*C* after spraying.

In many, but not all, prior empirical studies of insect populations, TL has been confirmed with *b* < 2. If *b* < 2, then 2 –*b* > 0 and hence *s*^2-*b*^ < 1. Then the coefficient *s*^2-*b*^*C* of TL after spraying should be smaller than the coefficient *C* of TL before spraying. On the other hand, if *b* > 2, then 2 –*b* < 0 and hence *s*^2-*b*^ > 1. The coefficient *s*^2-*b*^*C* of TL after spraying should then be larger than the coefficient *C* of TL before spraying. If *b* = 2, the coefficients before and after spraying should be identical.

Model 1 gives six testable predictions.

(1) For every habitat *h* = 1, 2, …, *H*, we have *E*(*A*(*h*)) = *sE*(*B*(*h*)). This equation relates population means before and after spraying. If we plot the sample mean relative population density of vectors in habitat *h before* spraying on the horizontal axis and the sample mean relative population density of vectors in habitat *h after* spraying on the vertical axis, the data points should fall approximately along a straight line through the origin with slope *s*, apart from sampling variability in both the horizontal and the vertical coordinates of each point.
(2) For every habitat *h* = 1, 2, …, *H*, we have *Var*(*A*(*h*)) = *s*^2^*Var*(*B*(*h*)). This equation relates population variances before and after spraying. If we plot the sample variance of relative population density of vectors in habitat *h before* spraying on the horizontal axis and the sample variance of relative population density of vectors in habitat *h after* spraying on the vertical axis, the data points should fall along a straight line through the origin with slope *s*^2^, apart from sampling variability in both the horizontal and the vertical coordinates of each point.
(3) Because we assumed 0 < *s* < 1, it follows that *s*^2^ < *s*, so the slope of the line for the sample variances of relative population density before and after spraying should be smaller (lower) than the slope of the previous line for sample means of relative population densities before and after spraying.
(4) The sample means and the sample variances of relative population density before and after spraying should both obey TL if either one does.
(5) The slope or exponent *b* should remain unchanged before and after spraying, apart from sampling variability in the estimates of *b*.
(6) The coefficient of the power-law form of TL should change from *C* before spraying to *s*^2-*b*^*C* after spraying. Apart from sampling variability in the estimates of *s* and the parameters of TL, this coefficient after spraying will be smaller than, equal to, or larger than the coefficient of TL before spraying according as *b* < 2, *b* = 2, and *b* > 2.

If model 1 is supported by data, then *s* provides a valuable statistical summary of the overall effectiveness of spraying. The smaller *s* is, the more effective the spraying was.

In testing prediction (1) above, if most of the data points (*E*(*B*(*h*)), *E*(*A*(*h*))), *h* = 1, 2, …, *H*, fall along a straight line *E*(*A*(*h*)) = *sE*(*B*(*h*)) but one or two points fall far above the line, then the control for the habitats corresponding to these outliers needs to be strengthened. If most of the data points (*E*(*B*(*h*)), *E*(*A*(*h*))), *h* = 1, 2, …, *H*, fall along a straight line but one or two points fall far below the line, then the control for the habitats corresponding to these outliers is unusually effective or the vector populations in those habitats are unusually vulnerable, and some control effort for these habitats might be directed to other more difficult habitats.

In testing prediction (2) above, if most of the data points (*Var*(*B*(*h*)), *Var*(*A*(*h*))), *h* = 1, 2, …, *H*, fall along a straight line *Var*(*A*(*h*)) = *s*^2^*Var*(*B*(*h*)) but one or two points fall far above the line, then spraying is not being uniformly applied to all sites of the habitats corresponding to these outliers or the vector populations are variably vulnerable to spraying for reasons that need to be determined (for example, because of differences in the physical complexity of sites considered to belong to the same habitat). If most of the data points (*Var*(*B*(*h*)), *Var*(*A*(*h*))), *h* = 1, 2, …, *H*, fall along a straight line but one or two points fall far below the line, then these habitats may lack established bug colonies. Further investigation would be required to determine whether such habitats have few adult bugs and the offspring cannot develop a new colony, leading possibly to Poisson variation among sites. This scenario is likely for the sylvatic triatomine species in certain peridomestic habitats.

#### Model 2: Random survival proportion after spraying

Suppose that the fraction of vectors that survive spraying at a particular site of habitat *h* is a random variable *S*(*h*) where 0 < *S*(*h*) < 1. Model 2 assumes that this survival fraction may differ among sites of each habitat *h* (e.g., may be different for every chicken coop in the study area) and has a distribution that depends on the habitat *h* (e.g., the distribution for chicken coops may differ from the distribution for goat corrals). Also assume that *S*(*h*) and *B*(*h*) are independent for all habitats *h* = 1, 2, …, *H*.

Then for every habitat *h* = 1, 2, …, *H*, the number of vectors that survive spraying at a particular site of habitat *h* is *A*(*h*) = *S*(*h*)*B*(*h*). Both sides of this equation are random variables with values that vary among the sites of habitat *h*. By the assumed independence between *S*(*h*) and *B*(*h*), the "product rule" holds: *E*(*A*(*h*)) = *E*(*S*(*h*)*B*(*h*)) = *E*(*S*(*h*))*E*(*B*(*h*)). Thus *E*(*B*(*h*)) = [*E*(*S*(*h*))]^-1^*E*(*A*(*h*)).

The variance of *A*(*h*) is more complicated in model 2 than in model 1. The Detailed Methods in S1 Text prove that *Var*(*A*(*h*)) = *Var*(*B*(*h*))*E*([*S*(*h*)]^2^) + *Var*(*S*(*h*))[*E*(*B*(*h*))]^2^, which may also be derived from a formula of Goodman (1960) for the variance of a product of random variables. The product rule for *E*(*A*(*h*)) and this formula for *Var*(*A*(*h*)) apply separately to each habitat *h*.

Model 1 is the special case of model 2 in which *S*(*h*) = *s* with probability 1 for all habitats *h* and for all sites of each habitat, where 0 < *s* < 1. In this special case of model 2, *E*(*S*(*h*)) = *s* and *E*([*S*(*h*)]^2^) = *s*^2^ and *Var*(*S*(*h*)) = 0. Then the product rule *E*(*A*(*h*)) = *E*(*S*(*h*))*E*(*B*(*h*)) reduces to *E*(*A*(*h*)) = *sE*(*B*(*h*)) and the second term of *Var*(*A*(*h*)) = *Var*(*B*(*h*))*E*([*S*(*h*)]^2^) + *Var*(*S*(*h*))[*E*(*B*(*h*))]^2^ becomes 0, leaving *Var*(*A*(*h*)) = *s*^2^*Var*(*B*(*h*)). These equations are precisely those derived above for model 1. The difference between models 1 and 2 in the variance of relative population density after spraying is the second term *Var*(*S*(*h*))[*E*(*B*(*h*))]^2^ contributed by the variation in *S*(*h*).

Now suppose the relative population density of vectors satisfies TL before spraying, namely, *Var*(*B*(*h*)) = *C*[*E*(*B*(*h*))]^*b*^ for *h* = 1, 2, …, *H*. (TL asserts that *b* and *C* do *not* depend on the habitat *h*.) Then the Detailed Methods in S1 Text prove that

$$Var\left(A\left(h\right)\right)=\left\{CE\left({\left[S\left(h\right)\right]}^{2}\right){\left[E\left(S\left(h\right)\right)\right]}^{\text{-}b}\right\}{\left[E\left(A\left(h\right)\right)\right]}^{b}+\left\{Var\left(S\left(h\right)\right){\left[E\left(S\left(h\right)\right)\right]}^{\text{-}2}\right\}{\left[E\left(A\left(h\right)\right)\right]}^{2}.$$

We also prove in the Detailed Methods in S1 Text that in model 2, if *b* = 2 and the coefficient of variation of *S*(*h*) (across sites of habitat *h*) is the same for every habitat *h*, then *Var*(*A*(*h*)) and *E*(*A*(*h*)) satisfy TL with *b* = 2. The assumption that *b* = 2 in TL before spraying means that the coefficient of variation (across sites) of relative population density is the same for all *h*.

Because the probability distribution (including its mean and variance) of the spraying survival fraction *S*(*h*) is assumed to vary from habitat to habitat, model 2 gives fewer testable predictions than model 1. Assume the means and the variances of relative population density before spraying obey TL.

(7) If the exponent of TL before spraying is *b* = 2 and the coefficient of variation of *S*(*h*) (across sites of habitat *h*) is the same for every habitat *h*, then the means and the variances of relative population density after spraying obey TL with the same exponent *b* = 2.

The ratios *E*(*A*(*h*))/*E*(*B*(*h*)) = *E*(*S*(*h*)), *h* = 1, 2, …, *H*, summarize the habitat-specific effectiveness of spraying. The smaller the value of *E*(*S*(*h*)), the more effective the spraying of habitat *h*.

In the limit as *Var*(*S*(*h*)) → 0, model 2 becomes a model intermediate between model 1 and model 2, in which the spraying survival *s*_*h*_ = *E*(*S*(*h*)) is the same for all sites of habitat *h* but varies from one habitat *h* to another.

## Results

With *m* the sample mean of the relative bug population density of each habitat with at least one infestation detected and *v* the corresponding sample variance, Table 2 gives the estimated intercept *a* and slope *b* of Taylor's law (TL) log_10_ *v* ≈ *a* + *b* × log_10_ *m*, the standard errors of *a* and *b*, the adjusted *R*^2^ and several associated statistical tests of the linear regression, and the minimum, maximum, and range (over all habitats with at least one infestation detected) of log_10_ *m*, for each study, vector species, intervention status, and survey (83 cases).

### Amamá

#### Testing Taylor's law

**Fig 2:**
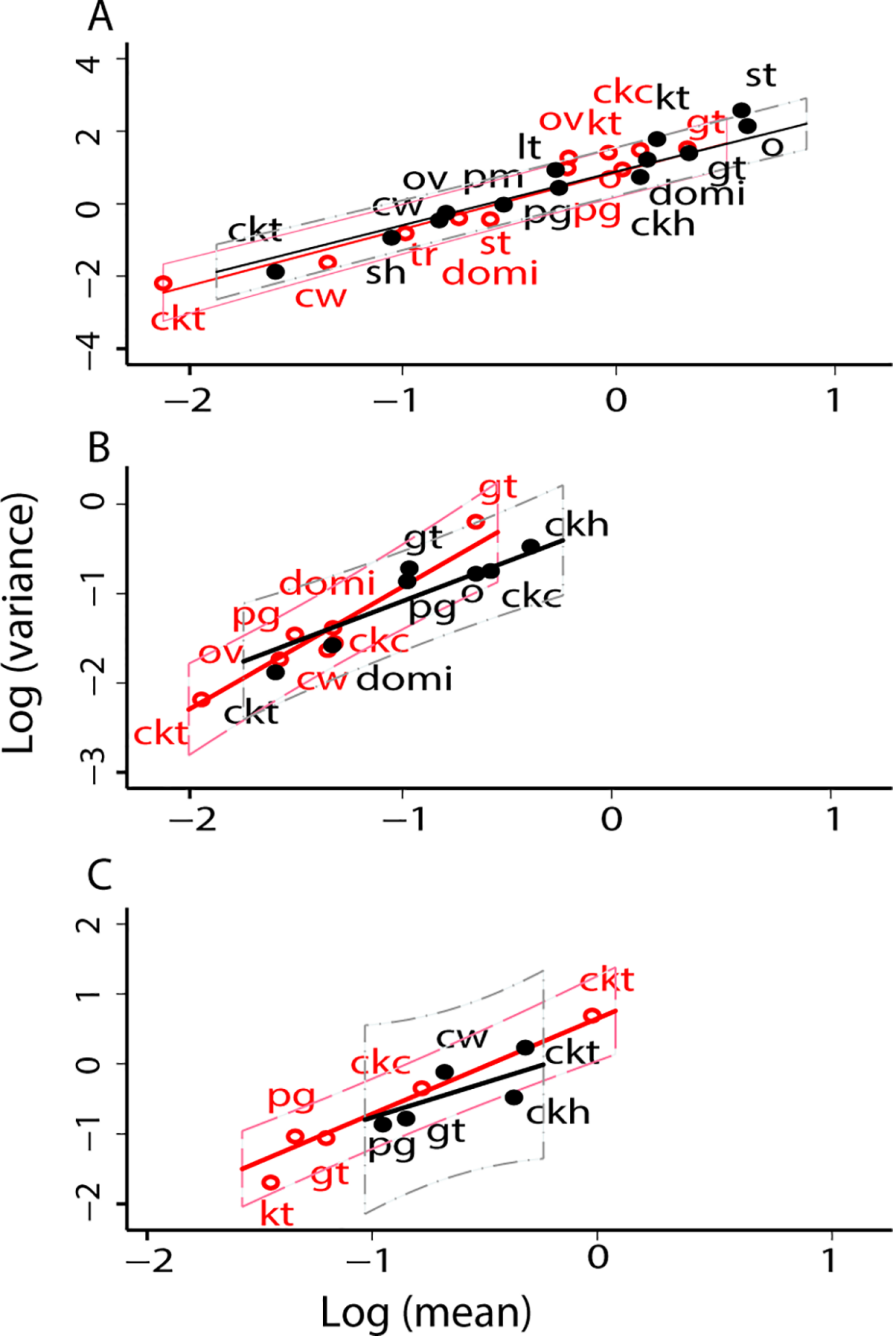
For rural communities around Amamá village [30], TL described the relationship between *y* = log_10_ *v* and *x* = log_10_ *m* of the relative abundance of *T*. *infestans* (A), *T*. *guasayana* (B), and *T*. *garciabesi* (C) in (peri)domestic habitats. Thirteen habitats in the periphery under pulsed control are represented by filled circles, and 11 habitats in the core under sustained vector surveillance and control are represented by open circles. Some habitats are repeated between core and periphery. Each point represents the mean and variance of bug abundance for one habitat. Key: ckt, tree with chickens; sh, open shed; ov, oven; pm, piled materials; o, other; cw, cow corral; lt, latrine/bathroom; pg, pig corral; ckc, chicken coop; ckh, small chicken house; kt, kitchen; gt, goat or sheep corral; st, storeroom. The solid straight lines represent the fitted regressions. The dashed belt above and below each solid regression line represents a 95% confidence interval for individual residuals. The red line is fitted to the core (open circles), the black, to the periphery (filled circles).

The data from Amamá confirmed TL. The log-mean relative abundance of *T*. *infestans* in (peri)domestic habitats was highly significantly correlated with the log-variance of relative abundance over the pooled core and periphery (*b* = 1.633 point estimate ± 0.089 standard error, *a* = 1.310 ± 0.082) (Fig 2A, Table 2). Residuals showed no significant deviations from normality, homoskedasticity and normal kurtosis. Adding a quadratic term to each of the equations did not significantly improve the fit of the models. Similar results held for *T*. *guasayana* and *T*. *garciabesi* in the data from the pooled core and periphery (Fig 2B and 2C, Table 2), except for marginally significant results for *T*. *guasayana* in the tests for heteroskedasticity and the information matrix, which could easily have been type 1 errors.

#### Comparing interventions

The Amamá area included two groups of rural communities that had different histories of insecticide control against *T*. *infestans*. The core had sustained control. The periphery had pulsed control. S1 Text describes the differences between the core and periphery in greater detail. The data from Amamá do not argue for or against Model 1 because neither region was before spraying or after spraying. Nevertheless, it is informative to compare the core and periphery by the same approach that we will use to compare the distribution of vectors before and after interventions in Olta, Figueroa, and Pampa del Indio.

ANCOVA found no significant differences in the slopes and intercepts of TL for *T*. *infestans* between the core (*b* = 1.610 ± 0.112, *a* = 1.070 ± 0.105) and the periphery (*b* = 1.576 ± 0.106, *a* = 1.080 ± 0.087), indicating no significant effects of the history of insecticide control on TL for this species (Table 2). However, different triatomine species had highly significantly different parameters of TL (Fig 2A–2C).

For *T*. *garciabesi* (Fig 2C), the mean and variance of abundance were larger under sustained control (core, open circle) than under pulsed control (periphery, filled circle), as illustrated for the main habitat of *T*. *garciabesi*, trees with chickens (ckt). The core had twice as many trees with chickens as the periphery. Moreover, inter-house distances between houses were lower and the proportion of houses raising chickens was higher in Amamá village than they were in rural villages of the periphery, deeper in the forest. Apparently these differences favored the apparently limited dispersal capacity of *T*. *garciabesi* in the core.

For Amamá, Fig A(A) in S1 Text gives, for each habitat (individual data points), the total number of *T*. *infestans* individuals and the mean (Fig A(B) in S1 Text) and the variance (Fig A(C) in S1 Text) of the number of *T*. *infestans* individuals per site of each habitat, in the core (horizontal axis) and in the periphery (vertical axis). We give two least-squares regressions for each summary statistic, and focus here on the regression through the origin, since in each panel the horizontal and vertical axes measure the same quantity under different conditions.

On the average across habitats, the total number of *T*. *infestans* in all sites of a habitat in the periphery was about 23% more than the total number of *T*. *infestans* in all sites of a habitat in the core (Fig A(A) in S1 Text). It cannot be concluded from this comparison that the pulsed surveillance in the periphery is inferior, as a method of reducing *T*. *infestans* populations, to the sustained surveillance in the core, because these data do not include a baseline of the initial *T*. *infestans* populations in the core and periphery prior to any control. Thus we cannot compare bug population sizes before and after control in the two regions.

The mean number of *T*. *infestans* per site of each habitat in the periphery was very loosely (adj. *R*^2^ = 0.37) related to the mean number of *T*. *infestans* per site of each habitat in the core (Fig A(B) in S1 Text). If there was any relation at all, the mean in the periphery was about twice as great as in the core.

Likewise, the variance of the number of *T*. *infestans* per site of each habitat in the periphery was very loosely, or hardly at all, related to the variance of the number of *T*. *infestans* per site of each habitat in the core (Fig A in S1(C) Text). Storerooms were the outlier in the upper left corner of the plot. If there was any relation at all, the variance in the periphery was about 64% greater than in the core.

The regressions above included only habitats that had a mean greater than 0 in both core versus periphery, to be consistent with the following analyses in Olta, Figueroa, and Pampa del Indio, which included only habitats that had a mean greater than 0 both before and after interventions. This constraint reduced the number of data points in the above regression lines. Including all 15 data points (regardless of whether one of the means was 0) reduced the no-intercept regression slope of the mean abundance in the periphery to the mean in the core from 2.0060 (± 0.7969) to 1.5825 (± 0.5928, adj. *R*^2^ = 0.290) and the slope of the variance from 1.6387 (± 2.9043) to 1.1610 (± 1.8592, adj. *R*^2^ = -0.042).

#### Core area longitudinal surveys

**Fig 3:**
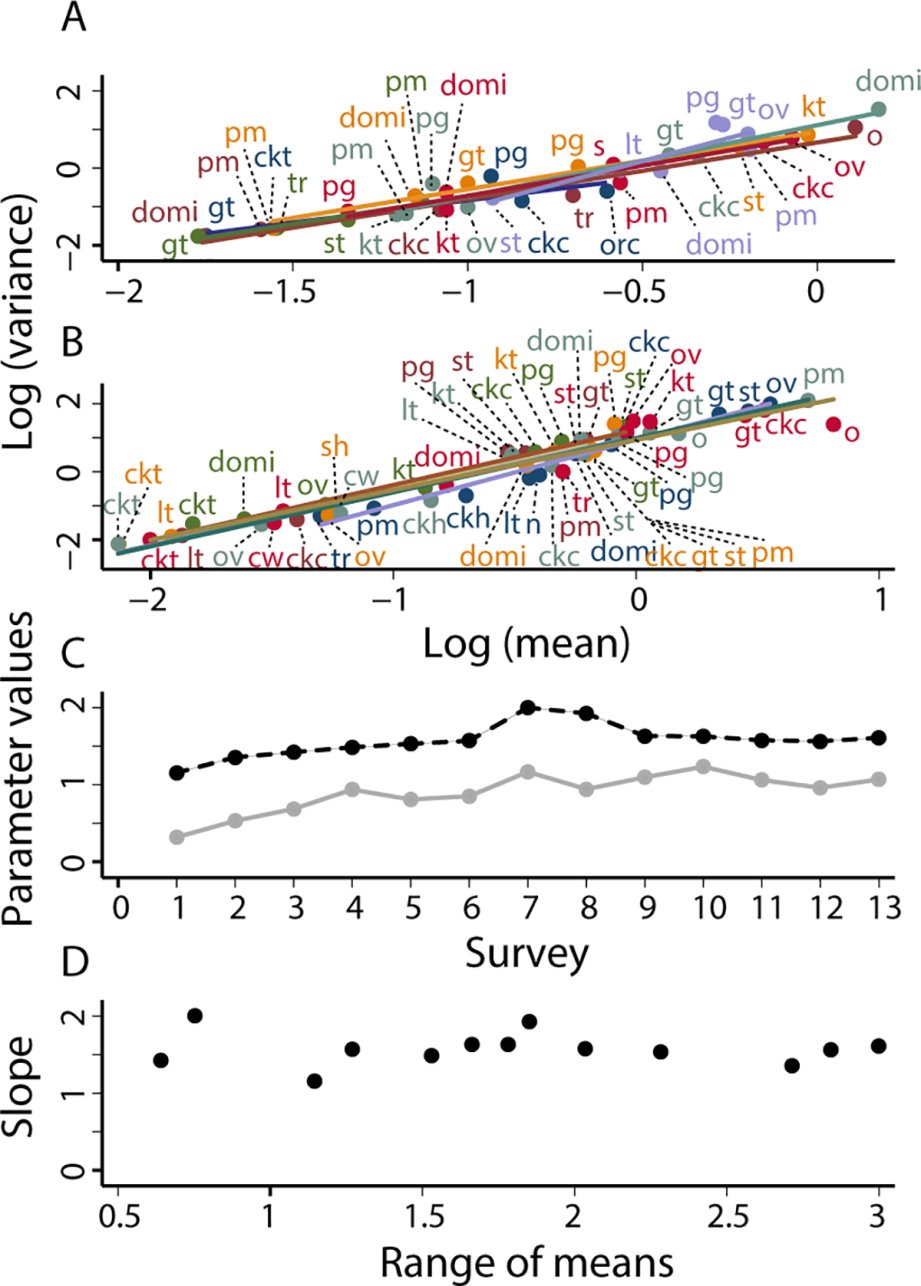
For *T*. *infestans* in the Amamá core under sustained vector surveillance and control, in (A) surveys 1 (October 1993) to 7 (May 1997) and (B) surveys 8 (November 1997) to 13 (October 2002), TL described the relationship between *y* = log_10_ *v* and *x* = log_10_ *m* of the relative abundance of *T*. *infestans*. Each point represents the mean and variance of bug abundance for one habitat at one survey. The solid straight lines are fitted by least-squares regression to the data from each survey separately. (C) Values of the parameters *a* (solid gray line) and *b* (dashed black line) of TL at each of the 13 surveys. (D) Slope *b* as a function of the range (maximum log_10_ mean minus minimum log_10_ mean) of the number of bugs. The greater the range, the smaller the variability in *b*. Key as in Fig 2.

In the 13 surveys from October 1993 to October 2002 of the Amamá core under sustained vector surveillance and control, the mean and variance of the relative abundance of *T*. *infestans* bugs in each habitat obeyed TL (Fig 3A and 3B, Table 2). Eleven of the 13 tests rejected the null hypothesis that *a* = 0, and all point estimates of *a* were positive. Of the 52 = 4 × 13 tests of various assumptions of the linear model, only 3 had *P* < 0.05, and 3/52 = 0.058 was close to the fraction 0.05 expected by chance alone. The linear model of TL was not rejected. For 10 of the 13 surveys, the adjusted *R*^2^ of TL exceeded 0.9. In general, TL was a plausible model. Results were similar for the two other species in the Amamá core (Table 2).

The intercept *a* and the slope *b* of TL fluctuated to some extent but displayed no systematic trend over time (Fig 3C, Table 2). The point estimate of the slope reached a peak of *b* = 2.002 ± 0.720 at survey 7 (May 1997). The greater the range (maximum log_10_ mean minus minimum log_10_ mean) of the mean numbers of bugs by habitat, the smaller the fluctuations of *b* around the central value of roughly 1.5 (Fig 3D, Table 2). Of the 13 estimates of slope, 10 fell between 1.4 and 1.65. Seasonal and secular changes during this decade had little effect on the form of TL or its parameters.

For the less abundant bug species, *T*. *guasayana* and *T*. *garciabesi*, the patterns were similar (Table 2), though with slightly more fluctuations.

### Olta

#### Testing Taylor's law

**Fig 4:**
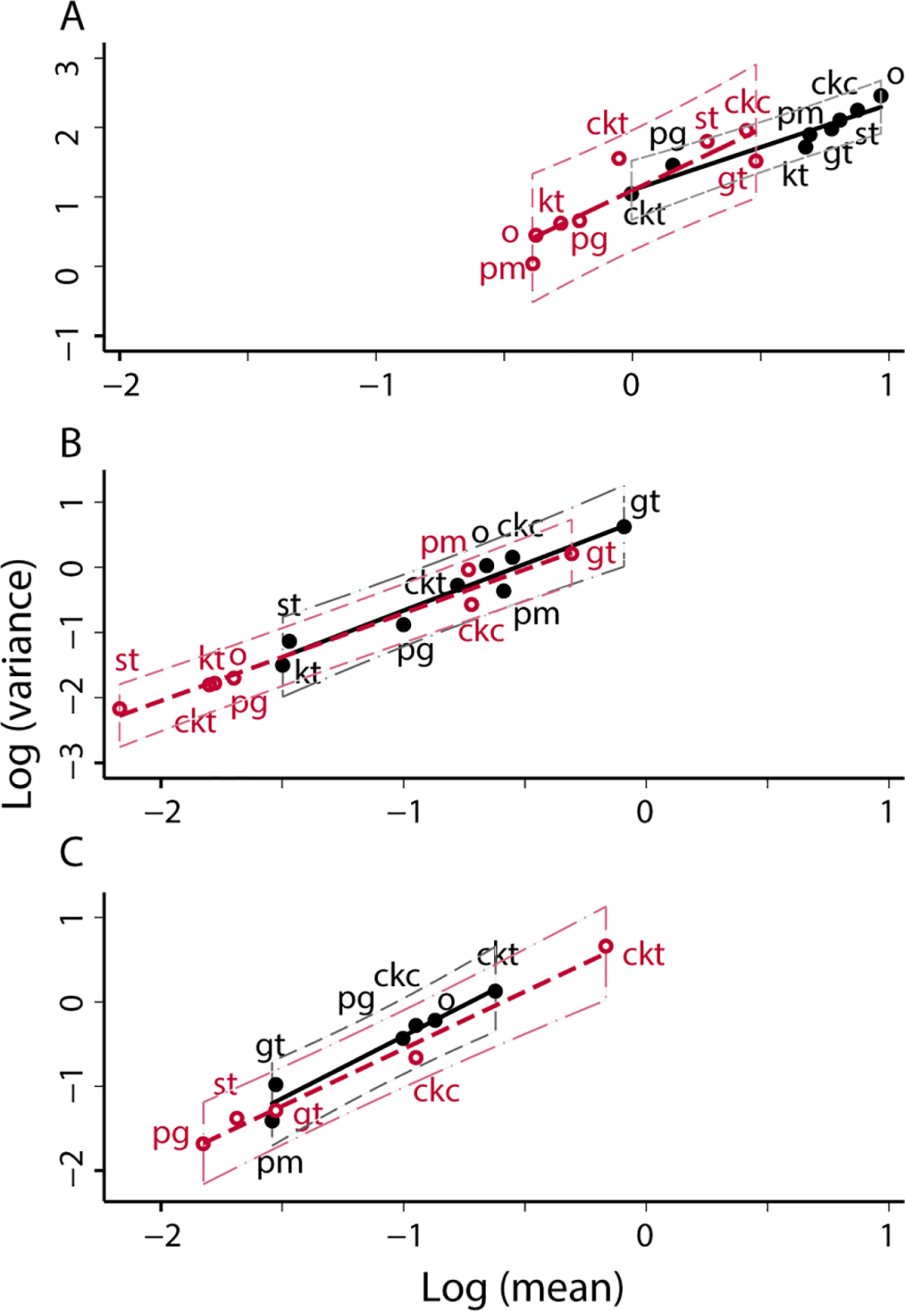
In Olta, 1999–2000 [33], TL described the relationship between *y* = log_10_ *v* and *x* = log_10_ *m* of the relative abundance of *T*. *infestans* (A), *T*. *guasayana* (B), and *T*. *garciabesi* (C) in 5–8 peridomestic habitats searched for bugs with equal catch effort per site, before (filled black circles) and one year after (open red circles) a community-wide spraying with insecticides. Each point represents the mean and variance of bug abundance for one habitat and bug species. The solid straight lines are fitted by least-squares regression to the data from each survey separately. Key as in Fig 2.

The data from Olta confirmed TL. Before community-wide insecticide application, the log-mean relative abundance of *T*. *infestans*, *T*. *guasayana* and *T*. *garciabesi* in peridomestic habitats was highly significantly correlated with the log-variance of bug abundance when the three species were taken together (adj. *R*^2^ = 0.981), with a slope (*b* = 1.504 ± 0.045) suggesting significant insect aggregation or differences in habitat suitability across habitats (Table 2). Approximately the same patterns were recorded when each species was taken separately before interventions: *T*. *infestans* (*b* = 1.233 ± 0.150; Fig 4A), *T*. *guasayana* (*b* = 1.428 ± 0.167; Fig 4B), and *T*. *garciabesi* (*b* = 1.558 ± 0.190; Fig 4C). All intercepts were significantly different from 0 whereas the quadratic term was not statistically significantly different from 0 for each triatomine species both before and after insecticide spraying (Table 2). Residuals showed weak deviations from normality, homoskedasticity and normal kurtosis for the three species.

Community-wide insecticide application did not destroy the linear relationship between the log-variance and log-mean of bug abundance for the three triatomine species taken together (*b* = 1.646 ± 0.240, *a* = 1.037 ± 0.083) (Table 2). The slopes of log-variance to log-mean bug abundance did not differ significantly among triatomine species either before or after insecticide spraying, nor when each species was compared separately before versus after spraying. Intercept values before and after insecticide spraying were not significantly different.

#### Effects of spraying: Testing predictions 1, 2, 3

**Fig 5:**
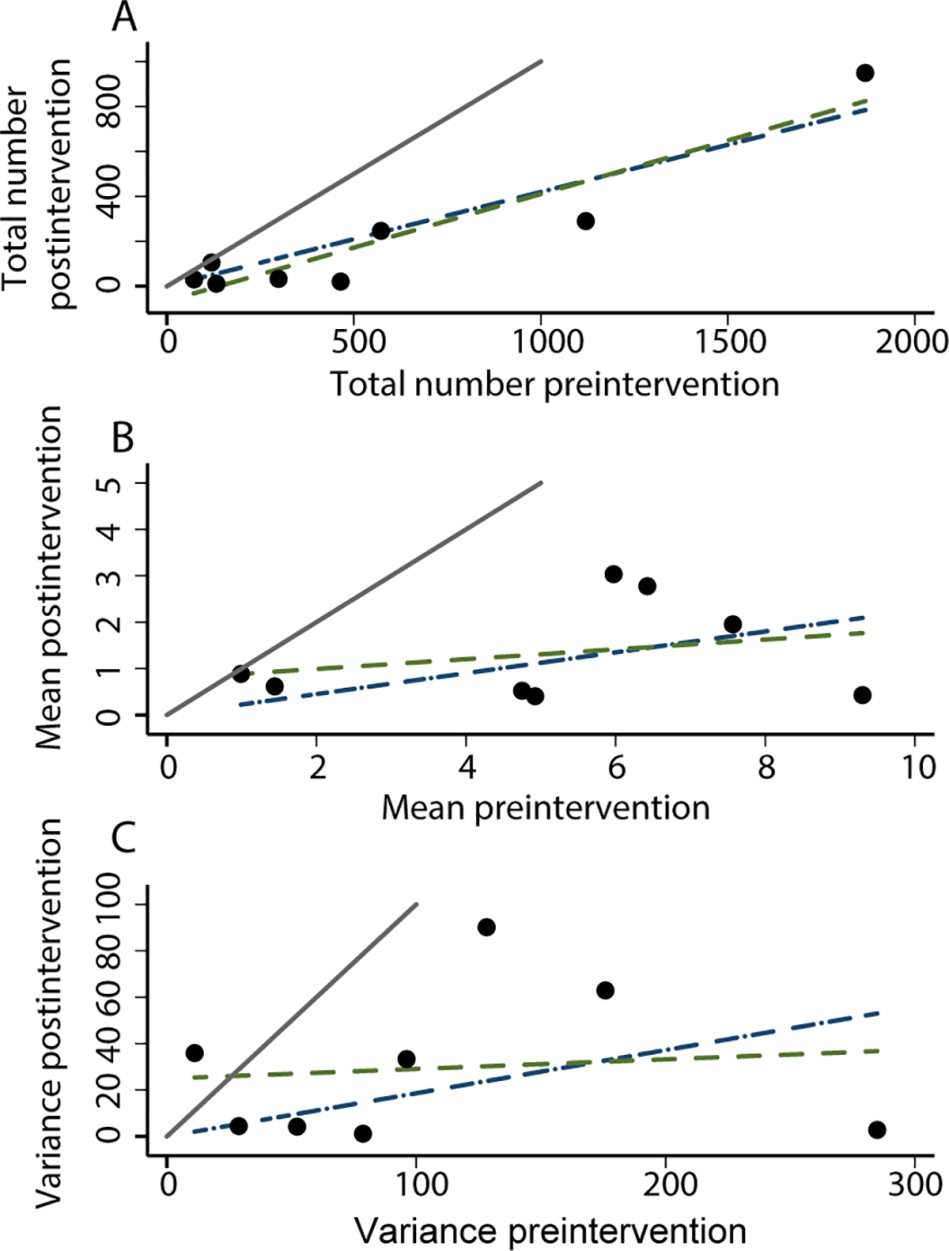
For Olta, the total (A), the mean number of *T*. *infestans* individuals per site of each habitat (B), and the variance of the number of *T*. *infestans* individuals per site of each habitat (C), before spraying (horizontal axis) and after spraying (vertical axis). In all panels, the thick solid line is the diagonal line of identity between vertical and horizontal axes, y = x. Two least-squares linear regressions allow an arbitrary intercept or require the line to pass through the origin (intercept = 0). The regression equations with (y = *b*x + *a*) and without intercept (y = *b*x) are (standard error within parentheses) for (A): y = 0.4763 (0.0728)x– 65.9622 (59.9026), adj. *R*^2^ = 0.8565, and y = 0.4196 (0.0523)x, adj. *R*^2^ = 0.888; (B): y = 0.1057 (0.1511)x + 0.7796 (0.8789), adj. *R*^2^ = -0.079, and y = 0.2250 (0.0681)x, adj. *R*^2^ = 0.554; (C): y = 0.0415 (0.1499)x + 24.9251 (20.3604), adj. *R*^2^ = -0.152, and y = 0.1860 (0.0956)x, adj. *R*^2^ = 0.259.

For Olta, Fig 5 gives, for each habitat (individual data points), the total (A), the mean (B), and the variance (C) of the number of *T*. *infestans* individuals per site of each habitat, before spraying (horizontal axis) and after spraying (vertical axis). The number of sites of each habitat was identical before and after spraying (S1 Table). We focus here on the regression through the origin, as above.

Both the mean (Fig 5B) and the variance (Fig 5C) after spraying were smaller than their values before spraying, as predicted by Model 1. They were loosely related to the mean and the variance (adj. *R*^2^ = 0.550, 0.259), respectively, before spraying, so predictions 1 and 2 of model 1 were weakly confirmed. The mean after spraying was approximately 23% of the mean before spraying, averaged over habitats by the linear regression. The variance after spraying was approximately 19% of the variance before spraying, averaged over habitats by the linear regression. Only for chicken trees was the variance after spraying greater than the variance before spraying (S1 Table). That the slope of the regression for the sample variances of relative population density before and after spraying was smaller than the slope of the regression for sample means of relative population densities before and after spraying confirms prediction 3 qualitatively. However, the data did not confirm the quantitative prediction that the slope of the variance regression should be the square of the slope of the mean regression. The slope of the variance regression, 0.186, substantially exceeded the squared slope of the mean regression, 0.225^2^ = 0.051.

#### TL and spraying: Testing predictions 4, 5, 6

The data from Olta (Table 2) confirmed prediction 4 (the means and the variances of relative population density before and after spraying should both obey TL if either one does) of model 1. For *T*. *infestans* and the other two vector species each considered separately and for all three species combined, both pre- and postintervention, there was no strong (*P* < 0.01) evidence to reject TL.

The data from Olta (Table 2) also confirmed prediction 5 (the slope or exponent *b* should remain unchanged before and after spraying). For *T*. *infestans*, the preintervention slope and standard error were *b* = 1.233, SE(*b*) = 0.150. The postintervention slope and standard error were *b* = 1.767, SE(*b*) = 0.347. The Welch test gave little or no evidence (*P ≈* 0.098) that the difference of slopes was significantly different from 0 (S3 Table). This conclusion is evident from inspection, since the difference between the pre- and postintervention slopes, 1.233–1.767 = -0.534 is not even twice the postintervention SE(*b*). The same holds for the other two vector species and for all three species combined.

The data from Olta (Table 2) also confirmed qualitatively prediction 6: the coefficient of the power-law form of TL should change from *C* before spraying to *Cs*^2-*b*^ after spraying. With *b* < 2, the coefficient of TL after spraying was smaller than the coefficient of TL before spraying. In Olta, for *T*. *infestans*, *b* < 2 both pre- and postintervention. As predicted, the preintervention *a* = 1.100 was (very slightly) larger than the postintervention *a* = 1.095. The same direction of difference held for the other two vector species individually. However, for all three species combined, the preintervention *a* = 0.995 was slightly (but not significantly) smaller than the postintervention *a* = 1.037.

### Figueroa

#### Testing Taylor's law

**Fig 6:**
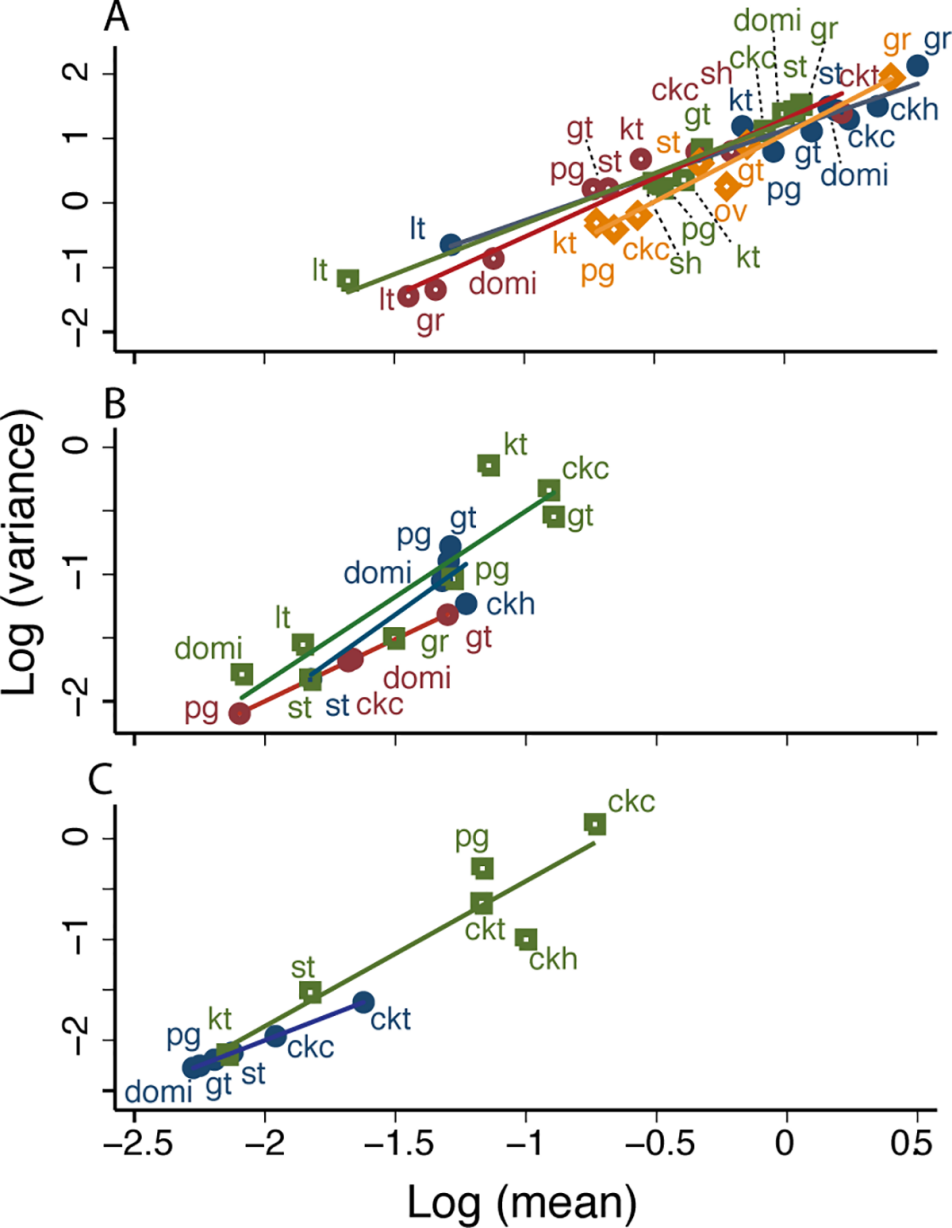
In Figueroa [34], TL described the relationship between *y* = log_10_ *v* and *x* = log_10_ *m* of the relative abundance of *T*. *infestans* (A), *T*. *guasayana* (B), and *T*. *garciabesi* (C) in 10 (peri)domestic habitats with positive mean abundance surveyed just before community-wide spraying with insecticides in October 2003 and during follow-up monitoring surveys of house/habitat infestations in which reinfested houses were selectively re-sprayed with insecticides in March and October 2004 and March 2005. Each point represents the sample mean and sample variance of bug abundance for one habitat on a specified date. The solid straight lines are fitted by least-squares regression to the data from each survey separately. Blue (solid circle), orange (diamond with white dot), red (circle with white dot), and green (square with white dot) points and lines represent October 2003, March and October 2004 and March 2005, respectively. Key to habitats as in Fig 2.

The data from Figueroa confirmed TL. The preintervention log-mean relative abundance of *T*. *infestans* in (peri)domestic habitats of Figueroa rural houses was highly significantly correlated with the log-variance of bug abundance (adj. *R*^2^ = 0.919). The slope *b* = 1.401 ± 0.146 suggested significant insect aggregation or differences in the variation of suitability across habitats (Table 2, Fig 6A). Residuals showed no significant deviations from normality, homoskedasticity and normal kurtosis. Mean bug abundance ranged over nearly two orders of magnitude. Bugs were very rare in latrines at the extreme left, which had very few bugs collected among the 115 sites inspected for infestation and only exceptionally had a bloodmeal host (chickens) inside or leaning against latrine walls. Latrines do not appear to harbor established bug populations. When both data points for latrines were suppressed, the point estimate of the slope (±standard error) rose to 1.494 ± 0.413 and adj. *R*^2^ fell to 0.634. Regardless of whether latrines were included or not, the confidence interval for the coefficient *c* of the quadratic term in the generalized TL included 0, indicating that the linear form of TL was not rejected by the data.

Community-wide insecticide spraying did not significantly affect TL for *T*. *infestans* except possibly at 5 months post-spraying (March 2004) (Fig 6A). Slopes for *T*. *infestans* increased from 1.401 ± 0.146 before spraying to 2.092 ± 0.225 at 5 months post-spraying (March 2004) (S3 Table gives *P* ≈ 0.016 for the Welch test of the difference in the slopes in October 2003 before spraying and 5 months later) and then decreased to 1.821 (*P* ≈ 0.033, in comparison with pre-spraying) and 1.562 (*P* ≈ 0.215, in comparison with pre-spraying) at 12 and 18 months post-spraying, respectively. Intercepts were significantly different from 0, and there was significant evidence of curvature at 12 and 18 months post-spraying (October 2004 and March 2005). Residuals showed no significant deviations from normality, homoskedasticity and normal kurtosis after spraying. For *T*. *garciabesi* and *T*. *guasayana*, the preintervention slopes of each did not differ significantly from the respective postintervention slopes (*P* > 0.10) (Fig 6B and 6C).

#### Effects of spraying: Testing predictions 1, 2, 3

For Figueroa, Fig B in S1 Text gives, for each habitat (individual data points), the total, the mean and the variance of the number of *T*. *infestans* individuals per site of each habitat, before spraying (October 2003) (horizontal axis) and after spraying (October 2004) (vertical axis). The number of sites of each habitat was substantially different before and after spraying (S1 Table). We eliminated three habitats in which the mean and variance were 0 before or after spraying (chicken trees, chicken houses, and open sheds), leaving eight habitats. We focus here on the regression through the origin, as above.

Both the mean (Fig B(B) in S1 Text) and the variance (Fig B(C) in S1 Text) after spraying are usually smaller than their values before spraying, as assumed by model 1. They are only loosely related to the mean and the variance, respectively, before spraying, so predictions 1 and 2 of model 1 are at best weakly confirmed. The mean after spraying was much smaller than that before spraying, approximately 9% of the mean before spraying. The variance after spraying was approximately 2% of the variance before spraying. That the slope of the regression for the sample variances of relative population density before and after spraying was smaller than the slope of the regression for sample means of relative population densities before and after spraying confirmed prediction 3 qualitatively. The quantitative prediction that the slope of the variance regression should be the square of the slope of the mean regression was (at best) roughly, or perhaps not, confirmed, as the slope of the variance regression, 0.0160, differed from the squared slope of the mean regression, 0.0928^2^ = 0.00861 by a factor of 2.

#### TL and spraying: Testing predictions 4, 5, 6

All three species observed in Figueroa (*T*. *garciabesi*, *T*. *infestans* and *T*. *guasayana*) satisfied TL before spraying (in the survey of October 2003), confirming prediction 4. Only for *T*. *infestans* do we have enough data to test TL in the first survey after spraying (March 2004), and for this species and survey, TL held (further confirming prediction 4).

For *T*. *infestans*, the preintervention slope *b* = 1.401 ± 0.146 was marginally significantly smaller than the immediate (March 2004) postintervention *b* = 2.092 ± 0.225, as noted above (*P* = 0.016) according to the Welch test (S3 Table, sheet WelchP). These observations of *T*. *infestans* are roughly consistent with model 1's prediction 5. By one year postintervention (October 2004), the slope for *T*. *infestans* was *b* = 1.821 ± 0.152 (*P* = 0.033 for the Welch test of the null hypothesis of no difference from the preintervention slope *b* = 1.401 ± 0.146). By 17 months postintervention (March 2005), the slope *b* = 1.562 ± 0.133 differed insignificantly (*P* = 0.215, S3 Table) from the preintervention *b*, again in agreement with model 1's prediction 5, but the quadratic term *c* differed significantly from zero (*P* = 0.006, Table 2), even though adj. *R*^2^ = 0.945.

The preintervention *a* = 1.129 ± 0.072 did not differ notably from the October 2004 postintervention *a* = 1.294 ± 0.127 or from the March 2005 postintervention *a* = 1.239 ± 0.083 (Table 2). These findings would be consistent with prediction 6 if spraying survival *s* were close to 1 in the subset of habitats with enough (here, usually 15 or more) infested sites after spraying to support calculations of the mean and variance.

For *T*. *guasayana*, the preintervention (October 2003) slope *b* = 1.468 ± 0.480 was larger, but not significantly larger, than the postintervention slope a year later (October 2004) *b* = 0.978 ± 0.010, *P* = 0.191 and the March 2005 slope *b* = 1.351 ± 0.255, *P* = 0.420, consistent with prediction 5 (Table 2).

### Pampa del Indio

#### Testing Taylor's law

**Fig 7:**
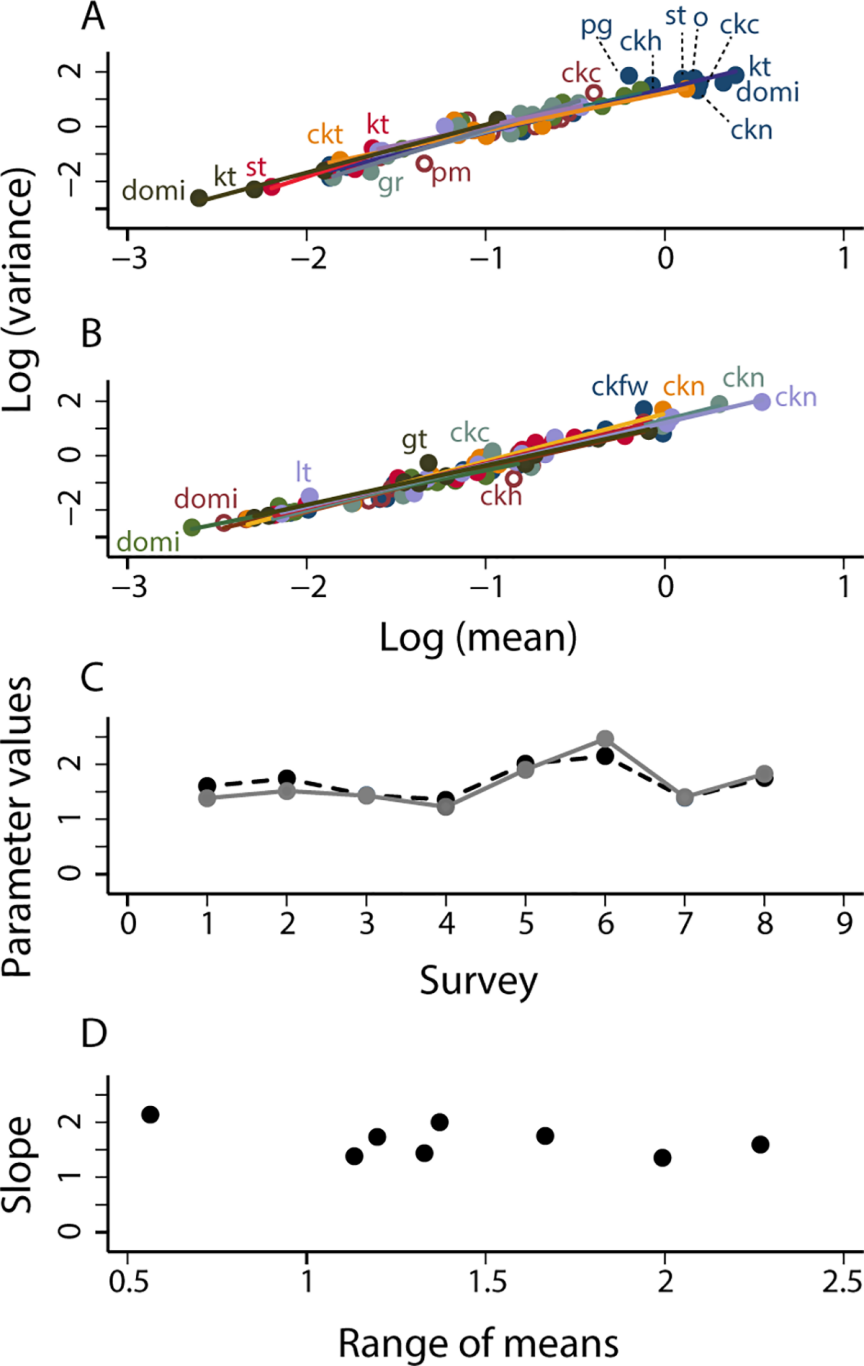
In Pampa del Indio [36], TL described the relationship between *y* = log_10_ *v* and *x* = log_10_ *m* of the relative abundance of *T*. *infestans* (A) and *T*. *sordida* (B) in 13 (peri)domestic habitats at survey 1 (October 2007, preintervention) to survey 8 (October 2010). Each point represents the mean and variance of bug abundance for one habitat and insect species. The solid straight lines are fitted by least-squares regression to the data from each survey separately. Key as in Fig 2. (C) Values of the parameters *a* (solid gray line) and *b* (dashed black line) of TL for *T*. *infestans* at each of the 8 surveys. (D) Slope *b* as a function of the range of the number of bugs for *T*. *infestans*. The greater the range, the smaller the variability in *b*.

The data from Pampa del Indio confirmed Taylor's law. The log-mean relative bug abundance in (peri)domestic habitats was highly significantly correlated with the log-variance of bug abundance before insecticide applications for *T*. *infestans* (*b* = 1.601 ± 0.112, *a* = 1.384 ± 0.105) and *T*. *sordida* (*b* = 1.671 ± 0.115, *a* = 1.249 ± 0.135) (Table 2, Fig 7A and 7B). The slopes suggest a similar degree of aggregation or diversity of habitat suitability across habitats and triatomine species. Intercepts were highly significantly different from 0. Residuals deviated significantly from homoskedasticity only for *T*. *sordida* according to the Breusch-Pagan/Cook-Weisberg test but not by Cameron and Trivedi's test. Adding a quadratic term to each of the equations did not significantly improve the fit of the models.

The slopes of TL postintervention varied more widely for *T*. *infestans* (range, 1.356–2.145) than for *T*. *sordida* (range, 1.316–1.748), possibly because the former had a very much reduced number of infested habitats over the last three surveys (Table 2). Intercepts postintervention varied from 1.224 to 2.460 for *T*. *infestans*, and from 0.778 to 1.542 for *T*. *sordida*. For *T*. *infestans*, the extreme high values of *a* and *b* occurred at survey 6 (October 2009) (Fig 7C) and were associated with the smallest range (0.564) between the maximal log_10_ mean of relative bug abundance and the minimal log_10_ mean of relative bug abundance (Fig 7D). These results illustrate the general pattern that estimates of the slope of any linear regression are unstable when the range of *x*-axis values is small. A larger range was generally associated with less variable point estimates of the slope. The eight surveys of Pampa del Indio made possible 64 = 8 × 8 pairwise comparisons between the two triatomine species of the slopes of TL. According to the Welch test (S3 Table), the slopes differed significantly between species only once with *P* < 0.01 and in only 7 of 64 comparisons with *P* < 0.05, giving no compelling evidence of difference in slopes between species. This detailed evidence for Pampa del Indio is consistent with the lack of strong evidence for a difference in slopes between these two species in Table 2.

#### Effects of spraying: Testing predictions 1, 2, 3

For Pampa del Indio, Fig C in S1 Text gives, for each habitat (each individual data point), the total, the mean and the variance of the number of *T*. *infestans* individuals per site of each habitat, before spraying (survey 1) (horizontal axis) and after spraying (survey 3) (vertical axis). The number of sites of each habitat was substantially different before and after spraying (S2 Table). We included here all habitats that had bugs before or after spraying, though we did not use all of these habitats to estimate TL (which requires nonzero means and nonzero variances). We focus here on the regression through the origin, as above.

Both the mean (Fig C(B) in S1 Text) and the variance (Fig C(C) in S1 Text) after spraying were smaller than their values before spraying, with the slight exception of latrines (S1 Table), and were visually linearly related (for the mean before and after, adj. *R*^2^ = 0.631, for the variance before and after, adj. *R*^2^ = 0.244), as predicted by Model 1. According to the linear regressions through the origin, the mean after spraying was approximately 13% of the mean before spraying. The variance after spraying was approximately 7% of the variance before spraying. That the slope of the regression for the sample variances of relative population density before and after spraying was smaller than the slope of the regression for sample means of relative population densities before and after spraying confirmed prediction 3 qualitatively. However, the quantitative prediction that the slope of the variance regression should be the square of the slope of the mean regression was at best weakly, or not, confirmed. The slope of the variance regression, 0.0664, exceeded the squared slope of the mean regression, 0.1253^2^ = 0.0157, by more than a factor of four.

**Fig 8:**
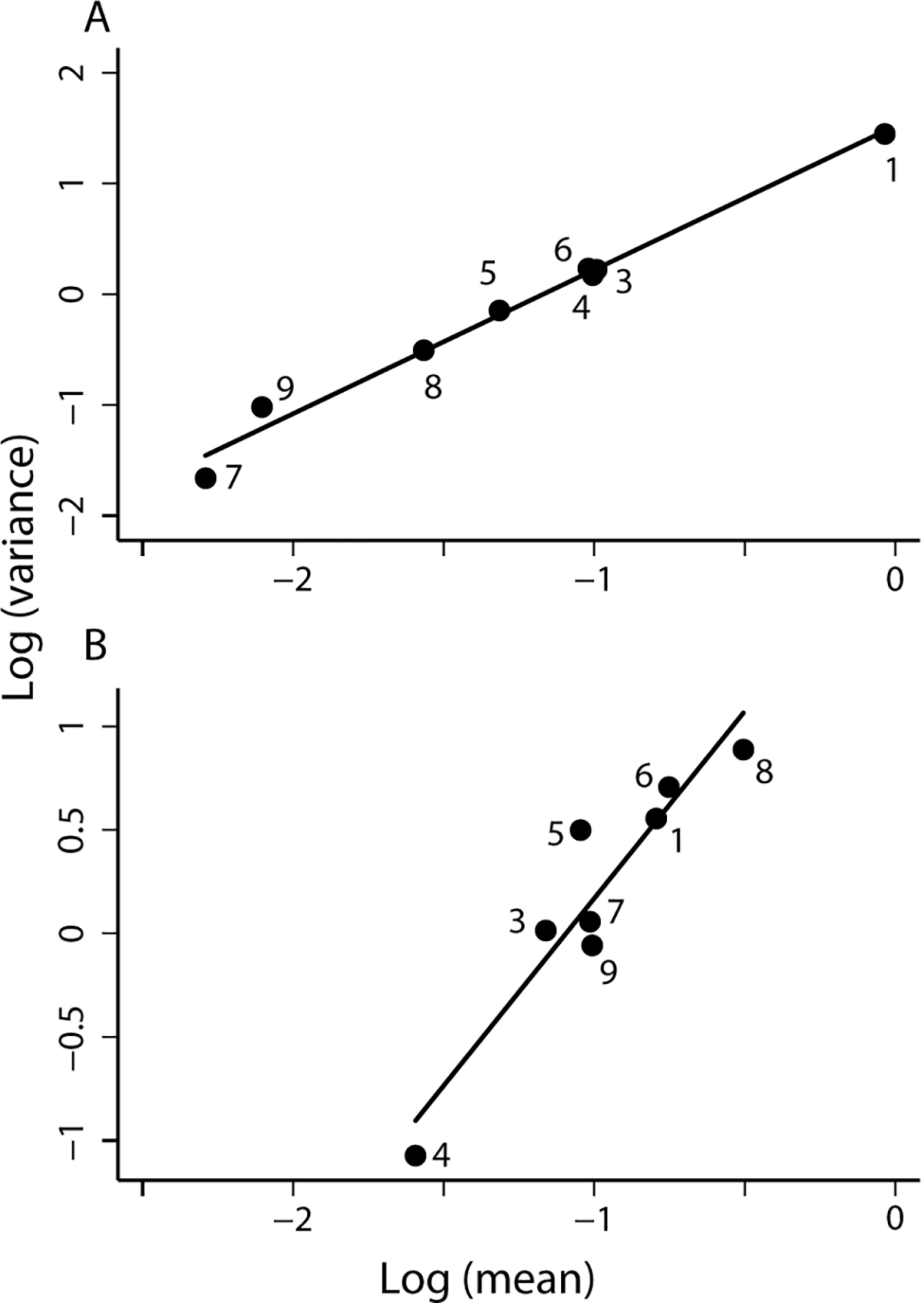
In Pampa del Indio [36], TL described the relationship between *y* = log_10_ *v* and *x* = log_10_ *m* of the relative abundance of *T*. *infestans* (A) and *T*. *sordida* (B) in (peri)domestic habitats before community-wide spraying with insecticides (survey 1, September-November 2007) and over the subsequent seven surveys (surveys 3 to 9) under selective insecticide applications of residual foci. (Survey 2 is not represented.) Each point represents the sample mean and sample variance (over all sites surveyed) of bug abundance for one survey and insect species, and the numbers give the survey number. The solid straight lines are fitted by least-squares regression.

For both *T*. *infestans* (Fig 8A) and *T*. *sordida* (Fig 8B), TL described well the relation of the log spatial sample variance to the log spatial sample mean of vector relative abundance across all habitats at each of eight periodic surveys conducted before (October 2007) and after (April 2008 to October 2010) community-wide insecticide application. During the three years after community-wide spraying, assessments of postintervention bug infestations were coupled with selective sprays of the residual foci detected except for April 2008. The linear relationship between the log-variance and log-mean of bug abundance for all sites and dates was highly significant for *T*. *infestans* (*b* = 1.299 ± 0.063, *a* = 1.592 ± 0.092) and *T*. *sordida* (*b* = 1.810 ± 0.267, *a* = 1.979 ± 0.275) (Table 2, Fig 8). Residuals showed either no or weak deviations from normality, homoskedasticity and normal kurtosis for both species. The slopes of TL differed marginally between triatomine species by the Welch test (*P =* 0.0559, S3 Table).

#### TL and spraying: Testing predictions 4, 5, 6

In Pampa del Indio, *T*. *infestans* and *T*. *sordida* satisfied TL before and after spraying (prediction 4). Though there was some heteroskedasticity preintervention in both studies, there was no statistically significant evidence of quadratic curvature (Table 2, Fig 7A and 7B).

For *T*. *infestans*, the preintervention (October 2007) slope *b* = 1.601 ± 0.112 did not differ significantly (*P* = 0.337, S3 Table) from the postintervention (April 2008) slope *b* = 1.738 ± 0.297, in agreement with model 1's prediction 5. The preintervention *a* = 1.384 ± 0.105 was smaller than, but did not differ significantly from, the postintervention *a* = 1.515 ± 0.276. The latter finding would be consistent with prediction 6 if spraying survival *s* were close to 1 in the subset of habitats with enough sites after spraying to support calculations of the mean and variance.

For *T*. *sordida*, the preintervention slope *b* = 1.671 ± 0.115 was larger, but not significantly larger, than the postintervention slope *b* = 1.508 ± 0.141, *P* = 0.190 (S3 Table), consistent with prediction 5. The preintervention intercept *a* = 1.249 ± 0.135 exceeded, but not significantly, the postintervention intercept *a* = 1.060 ± 0.200. The direction of the difference is qualitatively in accord with prediction 6 of model 1.

### Is the slope *b* of Taylor's law a fixed attribute unique to each vector species?

Taylor [3,4] estimated that different insect species had different slopes *b*. He proposed that the slope *b* was a characteristic unique and specific to each species, meaning that a given species always had the same value of *b* and no other species had that value of *b*. Others observed that Taylor had not considered the sampling variability in estimates of *b*. Two different species with identical population values of *b* could have different point estimates of *b* due to sampling fluctuations alone, not necessarily because of any statistically significant difference in *b* between them. Moreover, two different species with different population values of *b* could have identical or statistically indistinguishable point estimates of *b* due to sampling fluctuations alone. Moreover, Yamamura [45] and Cohen et al. [46] showed by examples that the physical scale on which population density was measured in a spatial TL could substantially affect the value of *b*, so that *b* was not necessarily an invariant species-specific characteristic.

The question of whether the slope *b* is a species characteristic (under some range of circumstances) has practical significance for Chagas disease vector control, because it implies further questions. For a given species, must *b* be measured anew in every new field situation? Must *b* be measured independently for different vector species?

S3 Table gives the Welch test statistic, df, and *P* for testing the null hypothesis of no significant difference between every two estimates of the slope *b* in Table 2 (excluding 4 of the 83 estimates, as we now describe). All these studies shared a similar ecology in the Gran Chaco and similar sampling methods. S3 Table and the following analyses omit two regression estimates in Table 2 from Amamá for *T*. *guasayana* in the core postintervention surveys of October 1993 and May 1999, for which df = 0 and 1, respectively, and the two estimates for Olta with three species combined, pre- and postintervention, leaving 79 = 83 - 4 species-specific estimates of *b*. There are 3,081 = 79×78/2 distinct pairs of estimates of *b*. Of these 3,081 comparisons, 206, or 6.7%, were less than 0.01, nearly seven times more than the approximately 31 = 3,081×0.01 *P* values less than 0.01 that would be expected on average by chance alone under the null hypothesis that all underlying values of *b* are identical.

Because each *P* value describes the difference of two slopes *b* and the same *b* occurs in multiple comparisons with all other values of *b*, we refrain from attempting to assign a probability to the chance that 3,081 dependent trials with marginal probability of success equal to 0.01 would yield 206 cases with *P* < 0.01 in the Welch test that two *b* values are equal. Instead, we simply infer that at least some of the differences in *b* were probably not due to sampling variation alone, and we focus attention on where the frequency of significant differences in *b* occurred most frequently.

Table 3 summarizes the number of cases where the Welch test's *P* < 0.01 for each pairing of each of the four species of *Triatoma* with itself and with each of the other three species. According to the diagonal elements of Table 3C, the percentage of *P* values that were less than 0.01 exceeded 1% by a factor of 2.8–4.7 when the slope for each of the four species was compared with the slope for the *same* species in another study. Each diagonal element was less than the percentage directly above it or directly to its right, meaning that there were fewer intraspecific than interspecific significant differences in slope. Each species resembled itself in TL slope *b* more than it resembled any other species, when we compared pairs of distinct studies. The largest percentages of *P* values that were less than 0.01 occurred in the comparison of *b* values of *T*. *guasayana* with those of the other three species. Overall, *T*. *guasayana* had *b* values that differed the most from those of the three other species, and overall the slope *b* of TL varied less within any of the four species than interspecifically. The *b* values from *T*. *sordida* and *T*. *garciabesi* hardly differed significantly more often interspecifically (3.2%) than they varied intraspecifically (2.8% for both species).

**Table 3:** Comparisons of TL slope *b* by *Triatoma* species (based on point estimates and standard errors in Table 2, summarizing results in S3 Table). (A) "Cell count" is the number of pairwise comparisons. For example, there were 9 values of *b* for *T*. *sordida* and 31 values of *b* for *T*. *infestans*, so there were 9×8/2 = 36 distinct intraspecific comparisons of *b* for *T*. *sordida*, 31×30/2 = 465 intraspecific comparisons of *b* for *T*. *infestans*, and 9×31 = 279 interspecific comparisons of *b* for *T*. *sordida* versus *T*. *infestans*. (B) "*P*<0.01 count" is the number of these comparisons that had *P* < 0.01 according to the Welch's test (S3 Table). (C) "%*P*<0.01" is the percentage of comparisons with *P* < 0.01. For example, for the comparisons of *b* for *T*. *sordida* versus *T*. *infestans*, 3.2% = 9/279. If differences in *b* were due to sampling fluctuations alone and there were no intra- or inter-specific differences in the underlying values of *b*, then "%*P*<0.01" = (*P*<0.01 count)/(Cell count) should approximate 1.0%.

(A) Cell count	*T*. *garciabesi*	*T*. *guasayana*	*T*. *infestans*	*T*. *sordida*
*T*. *garciabesi*	190	380	620	180
*T*. *guasayana*	0	171	589	171
*T*. *infestans*	0	0	465	279
*T*. *sordida*	0	0	0	36
(B) Number of P values < 0.01	*T*. *garciabesi*	*T*. *guasayana*	*T*. *infestans*	*T*. *sordida*
*T*. *garciabesi*	9	26	36	10
*T*. *guasayana*	0	5	73	24
*T*. *infestans*	0	0	13	9
*T*. *sordida*	0	0	0	1
(C) % of P values < 0.01	*T*. *garciabesi*	*T*. *guasayana*	*T*. *infestans*	*T*. *sordida*
*T*. *garciabesi*	4.7	6.8	5.8	5.6
*T*. *guasayana*	0	2.9	12.4	14.0
*T*. *infestans*	0	0	2.8	3.2
*T*. *sordida*	0	0	0	2.8

## Discussion

We review our most important empirical findings, discuss the findings in light of our mathematical models, suggest potential practical applications, and indicate some future research.

### Taylor's law describes the spatial distribution of Chagas disease vectors among habitats

With remarkable precision, Taylor's law (TL) described the relationship, among multiple habitats, of the variance of relative bug population density in sites of a given habitat to the mean of relative bug population density in the same sites of the given habitat. The log variance was well approximated as a linear function of the log mean in four studies that differed in geographic location, methods of bug control, time since spraying, degree of insecticide resistance, and principal bug species. The adjusted *R*^2^ had median 0.94 and lower and upper quartiles 0.91, 0.96 in 79 single-species field surveys with df > 1 in Table 2. The slope *b* of the linear relationship of log variance to log mean differed most between *Triatoma guasayana* and the other three species, *T*. *infestans*, *T*. *sordida*, and *T*. *garciabesi*. Interspecific differences exceeded intraspecific differences in slope *b*.

Slopes of TL generally, but not always, rejected Poisson-distributed vectors with different means in different habitats and were consistent with substantial spatial aggregation or differences in habitat suitability: the median slope *b* was 1.48 and the lower and upper quartiles were 1.35, 1.63 in these 79 field surveys (Table 2). Only four of these field surveys had *b* > 2, and none of these slopes was significantly greater than 2. Only four of these field surveys had *b* < 1, and none of these slopes was significantly less than 1. Thus none of the 79 TL field surveys significantly rejected the general pattern that 1 < *b* < 2, as has been widely found in other studies. The first inequality, 1 < *b*, implies that, when different habitats were compared, the variance in their bug populations *increased* faster than in proportion to their mean bug populations. The second inequality, *b* < 2, implies that, when different habitats were compared, the coefficient of variation (standard deviation divided by mean) *decreased* as the mean number of bugs per habitat increased.

### Models of control

Model 1 usefully highlights deviations from the predictions that follow from its very simple assumptions. The *increased* slope of TL for *T*. *infestans* observed after spraying in Olta, Figueroa, and Pampa del Indio indicates that spraying lowered mean vector abundance in some habitats more than in others, and hence was not uniformly effective across habitats. This heterogeneity in the effectiveness of spraying is also evident in the plots of log-mean-abundance-after-spraying as a function of log-mean-abundance-before-spraying (Fig 5, Fig B in S1 Text, Fig C in S1 Text). This evidence rejects the classic assumption made by vector-control agencies that insecticide spraying is uniformly effective. Heterogeneity in the effectiveness of spraying calls for targeted, improved vector control, possibly including appropriate environmental management measures. This issue is of paramount importance for the goal of large-scale Chagas disease vector elimination under the aegis of regional intergovernmental programs such as the Southern Cone and Central America Initiatives created in the 1990s and ongoing [47,48].

Model 1 has relevance beyond attempts to control Chagas disease vectors. As part of a study of the ecological consequences of sudden oak death in northeastern United States temperate forests, oak trees were censused and measured in 12 plots in 2007 and again in 2010 in Black Rock Forest, Cornwall, New York. Cohen et al. [49] fitted TL successfully to the counts of oak trees in 2007 before a major intervention in 2008 (killing of some oak trees by girdling) and again in 2010. The girdling of the oaks was analogous to spraying the bugs. The acceptable fits of TL before and after intervention were consistent with prediction 4 of model 1, and the absence of statistically significant evidence of a change in slope as a result of the intervention was expected from prediction 5 of model 1. Cohen et al. [49] offered no explanation of why the intervention did not significantly change the slope or destroy the fit of TL. Model 1 offers an explanation, or at least a phenomenological description.

### General implications and potential practical applications

Taylor's law identifies key habitats with high mean and variance of infestation before or after insecticide spraying. TL has not previously been used to discover highly variable infestations by any insect vector, to the best of our knowledge. In general, a habitat with exceptionally high variance, given its mean, would likely have a high likelihood of a vector (here bug) outbreak, and might deserve special attention for control by spraying or environmental alteration. A habitat with exceptionally low variance, given its mean, would be a habitat with a relatively stable endemic bug population. For example (Fig 2A), the spatial variance of *T*. *infestans* in chicken houses (ckh) in the periphery of Amamá is at or very near the lower limit of the 95% CI given the rather large spatial mean abundance of bugs in chicken houses in the periphery. Even with no spraying of insecticides, one might expect chicken houses with relatively stable chicken populations to harbor spatially consistent bug populations.

TL is useful for practical control efforts also for habitats that are not outliers from the log-log regression. For example, in the graphs of TL based on *T*. *infestans* abundance by habitat in Amamá, the data points at the upper right of the graph were granaries and sheds (i.e., open sheds with a thatched roof, frequently used as a storage area, with wide variation in stored contents and usage between households, sometimes with chickens nesting there). Overlooking or failing to inspect or treat adequately granaries and sheds could have large effects on bug persistence and subsequent house reinfestation rates. People rarely permit insecticide spraying of granaries with stored corn (except when the granaries are empty). The preintervention mean bug population size of granaries was large in Figueroa and a high proportion of granaries was infested [2]. Granaries were again at the top (upper right) of the log-log regression before and 5 months after spraying in Figueroa. Open sheds do not get much attention as potentially important habitats for bug control, perhaps because of the wide variability in bug counts between individual sheds. Granaries and open sheds had greater means and variances of bug abundance than chicken coops, storerooms, and domiciles. The latter three habitats usually take all the attention of bug control personnel. Domiciles, storerooms and kitchens tend to appear on the upper end of the log variance-log mean regressions. Because they concentrate competent hosts, these three habitats concentrate nearly all of the *T*. *cruzi*-infected bugs in any study area in the Gran Chaco, especially domiciles where all human-vector contacts occur (e.g., [50]).

The same argument applies to other habitats that receive less attention because they are located outside of domestic compounds in areas less disturbed by human activities. Examples include orchard fences where cavies and other rodents thrive and chickens sometimes nest [51], and abandoned constructions covered by vegetation and used by free-ranging goats, sheep or other domestic or sylvatic mammals [52]. Such areas are very rarely inspected for bugs or sprayed by bug control staff on the assumption that they are rarely infested (i.e., implicitly assuming that such habitats have an exceptionally large variance of bug population density) and because substantial time, effort and insecticide would be needed to spray them rigorously. Over the years, we collected several examples of such highly infested, rarely detected, habitats that would fall in the upper right corner of the graph of TL and would severely hamper any serious elimination campaign.

In the practical control of Chagas disease vectors, researchers and bug control people historically have shown little interest in levels of statistical precision or in using sampling theory. In the case of *T*. *infestans*, perhaps one reason was that the main initial program goal was to eliminate the vector completely from most of its range, not to control it or estimate its relative population density, and to treat all infested houses completely and homogeneously. Another possible reason was the crude method of detecting or sampling bugs, which is still used. Bug control programs declare house compounds infested or not. Though they may count the number of bugs per unit of search effort at the level of the house compound (distinguishing domestic from peridomestic habitats), they do not use this information other than for saying "many bugs or few bugs" at the community-wide level. Failure to use sampling theory in past practice does not diminish the present and future need to consider TL and its implications for certifying the interruption of transmission of *T*. *cruzi* to humans at a district- or state-wide scale, for example.

Figs 2–4, 6 and 7 make very clear which habitats have the largest means and variance of bug populations and how habitats respond to insecticide applications. Sometimes a habitat stays at the extreme right (with large mean and large variance of relative population size) after interventions while other habitats jump to the left extreme or remain there before and after interventions. That a habitat stays at the upper extreme after insecticide applications demonstrates the vulnerability of vector control through insecticides and the need for better chemical control or alternative interventions.

The habitat "other" appears several times with high mean abundance and high variance. Because "other" habitats are hard to classify, they may not be identified as important for insecticide applications. This problem is particularly acute for *Triatoma dimidiata* and other species where selective control is frequent (i.e., only domiciles, or only domiciles and chicken coops, are sprayed, for example). Neglected "other" habitats may be sources of recurrent infestations.

This collection of surveys and trials shows that insecticide-based vector suppression in several study areas across the Argentine Chaco has been far from uniformly successful over the 28 years from 1993 to 2010. The graphical display of means and variances by habitat may provide useful quantitative guidance for improving insecticide-based vector suppression.

### Future research

The relative population densities of Chagas vectors reported here strongly confirm TL but do not identify the mechanisms that may generate TL, such as insect behavior versus habitat suitability (measured by bug birth rates and death rates in different habitats). How important are selective dispersal and migration (insect behavior) versus differences between habitats in their average suitability for bugs and in their variation (across sites) of suitability? Control measures that transitorily lower the suitability of a habitat, such as residual insecticide spraying, could in principle stimulate adaptive behavioral responses by insects (e.g., excito-repellency), which may reduce the effectiveness of spraying at the level of the house compound or the village. Thus understanding the mechanisms that produce TL is important.

In these studies, postintervention surveys occurred from 4–6 months to 12 months after insecticide spraying. Hence the observed relative bug population density postintervention included immigration, local recruitment (new births of bugs), and local survival postintervention. Models 1 and 2 of habitat-specific persistence represent only the last of these processes, and therefore cannot be expected to account precisely for our field observations.

Models 1 and 2 assume that insecticide spraying does not reduce to zero the average or the variance of relative population density in sites of a habitat. They are models of successful partial control but not models of successful local elimination of bugs from all sites of any habitat. When the ultimate program goal of insecticide spraying is local elimination (suppression) of bugs from all sites of a habitat rather than control, these models may be helpful in identifying less vulnerable habitats using TL.

It would be useful to supplement these models with an empirical summary and theoretical model of the fraction of sites of each habitat from which spraying eliminated the vectors. If the number of bugs per site of a habitat were described by the negative binomial distribution, then the mean and variance would imply a fraction of sites with zero bugs. Efforts have been made to link TL with the mean and the variance of the negative binomial distribution [8,52–57], but it has recently been recognized that TL cannot hold with constant parameters at the same time that the negative binomial distribution holds with a constant scale parameter and a changing probability parameter ([9], p. E50) (see S1 Text for further details). A practically important topic for further research is finding a useful way to estimate the fraction of sites of a habitat with no bugs, when the mean and variance of bug abundance obey TL, as here.

The units of analysis in this paper are habitats such as chicken coops, kitchens, or granaries. By contrast, vector control programs use house compounds as units to calculate village-wide infestation rates. Additional empirical and theoretical analyses are needed to link habitats with house compounds and village-level summaries of infestation. Moreover, since the goal of vector control is to reduce or eliminate human *T*. *cruzi* infections, it would also be highly desirable to explore the potential of TL to shed light on the distribution of *T*. *cruzi* infection in vectors, humans and other animal hosts.

## Supporting information

S1 Text Contains detailed methods regarding data, statistical analyses, and models (including mathematical proofs of two variance formulas for model 2), detailed results, and four supplementary Figs A-D.
(DOCX)

S1 Table A key to habitat abbreviations and, for *Triatoma infestans* only, the sample means and sample variances of relative bug population density, by study, by survey, and by habitat. S1 Table includes no survey 2 for Pampa del Indio.
(XLSX)

S2 Table Raw and summary data of the studies from four areas. (XLSX)

S3 Table Comparisons of TL slope *b* for every pair of values in 79 studies, by area, *Triatoma* species, and intervention. Sheet "WelchT" gives the t-statistic of the Welch test. Sheet "Welchdf" gives the degrees of freedom (df) of the Welch's test. Sheet "WelchP" gives the P-value associated with the corresponding t and df.
(XLSX)
