# Supplementary material for: Chagas disease vector control and Taylor's law
Source: PLoS Negl Trop Dis. 2017 Nov 30;11(11):e0006092. doi: 10.1371/journal.pntd.0006092 (PMC5734788; doi:10.1371/journal.pntd.0006092)
Supplement: S1 Text — Contains detailed methods regarding data, statistical analyses, and models (including mathematical proofs of two variance formulas for model 2), detailed results, and four supplementary Figs A-D. (DOCX) [file pntd.0006092.s001.docx]

# Detailed Methods

## Field data

The data come from four large research projects in the Argentine Chaco region where Chagas disease was endemic. These projects aimed primarily to control the major vector *Triatoma infestans*, but also included observations of other local triatomines not considered as the main control targets. The surveys were conducted in well-defined rural areas of Olta (municipalities of General Belgrano and Chamical, La Rioja, western Argentina), Figueroa and Amamá (Figueroa and Moreno departments, respectively, Santiago del Estero, northwestern Argentina), and Pampa del Indio (General San Martín department, Chaco, northeastern Argentina). The study areas had been last sprayed with pyrethroid insecticides by vector control personnel from the Argentine federal or provincial vector control programs approximately 3 (Figueroa), 5-6 (Olta) and 11 years (Pampa del Indio) before our baseline assessments of house infestation, with no further control interventions in the intervening period.

The four studies shared similar vector survey methods aiming at full coverage of all house compounds and all potential bug habitats at each area. Bug counts included first- to fifth-instar nymphs and adults of any triatomine species collected. *Triatoma infestans* almost exclusively occurs in human sleeping quarters and peridomestic habitats (i.e., sylvatic foci are rare in the Argentine Chaco) whereas *Triatoma guasayana*, *Triatoma garciabesi* and *Triatoma sordida* have widespread sylvatic foci from which they invade and establish peridomestic colonies (i.e., with exceptional domestic foci in this region). In different study areas, different sets of habitats (chicken coops, goat corrals, etc.) were identified and surveyed. S1 Table gives a key to 21 of these habitats.

The spraying procedures were uniform in technique and coverage, but not in gear or pyrethroid insecticide dose, in Olta, Figueroa and Pampa del Indio where spraying was delivered by government teams. Government spraying covered all accessible structures, whether human-occupied or not, and whether or not they were known to be infested. Blanket sprays conducted immediately after baseline surveys of house infestation included all structures, regardless of whether they had been infested. This was standard procedure for the attack phase in rural areas, when house infestation prevalence with *T. infestans* exceeded 5%. (Spray coverage may differ in peri-urban or urban areas because of the great number of houses to be treated.) Right after the attack phase, during the surveillance phase, insecticide sprayings were selective and generally only included infested house compounds (not infested structures only but all the structures in the whole compound). Again, these were standard procedures for Chagas vector control programs. In actual practice (not in our insecticide trials), there was variation.

Government spraying differed from that in Amamá, the only area mainly under long-term, community-based vector surveillance and control, with selective pyrethroid treatments of reinfested houses and differing levels of monitoring of house infestation and supervision of control actions (details below). Community-based vector control was much more relaxed, covering infested house compounds only, but perhaps not all structures in a compound.

We now describe the four studies in greater detail, in the same order in which we shall present the results. In the data from Amamá, we compare two groups of villages with differing histories of vector control, and we also follow one of those two groups of villages longitudinally over 13 surveys during a decade. Then we compare different triatomine species before and after insecticide spraying in Olta (only one year after spraying), Figueroa (5-17 months after spraying, including a total of 4 rounds of vector surveillance plus immediate spraying) and Pampa del Indio (spatial and temporal averaging, every 4-5 months after spraying for a total of 8 rounds of vector surveillance plus immediate spraying).

### Amamá

The Amamá study included a core area with systematic control and a peripheral area with pulsed control. The core contained 5 villages (Amamá village, Trinidad, Mercedes, Villa Matilde and Pampa Pozo, all within 8 km from Amamá) and 137 houses as of October 2002 (Table 1). The core was under systematic vector surveillance and control in thirteen surveys from October 1993 to October 2002, as described previously (Gürtler et al. 2007). Detailed data on bug infestations by peridomestic habitat are not available prior to 1993. In the Amamá core, the only government-sponsored, community-wide campaigns of house spraying with insecticides ever conducted took place in October 1985 (Amamá village only) and 1992.

The periphery, which contained 35 villages and 186 houses, was sprayed with insecticides by householders in sporadic pulses over 1999-2001 as part of Plan Ramón Carrillo operations (Cardinal et al. 2007, Vazquez-Prokopec et al. 2009), and was first surveyed for bug infestation in May 2003. The periphery was more rural than the core: houses were deeper in the dry forest and less clumped, on average. The periphery had no prior government-sponsored insecticide campaign, unlike the core.

The intensity of bug infestations in core and periphery was determined by skilled vector control personnel who used timed manual collections with a dislodging aerosol (0.2% tetramethrin) and a fixed catch effort per site. Our data permit a longitudinal analysis of the core and a comparative analysis of the core and periphery.

In the core, only house compounds (domiciles and peridomestic structures) known to be infested (as determined by the research team or householders) were sprayed by vector control personnel supervised by the research team (between October 1993 and December 1997), and by a local reference person in charge of vector control who received variable degrees of assistance from householders (from May 1998 to October 2002); in fewer cases, householders directly sprayed their premises and reported the results to the local referent. Similar procedures were followed in the periphery, but there was neither external supervision of householders’ insecticide applications nor systematic monitoring of house reinfestation as in the core (Vazquez-Prokopec et al. 2009).

The prevalence of house infestation with *T. infestans* in domestic and peridomestic habitats of the core (11.7% and 31.3%, respectively) in October 2002 was substantially lower than in the periphery (17.6% and 50.7%, respectively) in May 2003 (Cardinal et al. 2007) (Table 1). All sites individually searched for triatomine bugs were classified into 17 (peri)domestic habitats on the basis of their structure, function, and main resident host (e.g., chicken, goat, human). Of 7,470 sites inspected in the core area between 1993 and 2002, 10.5% were infested with *T. infestans* (total catch, 2,067 bugs). Thirteen (periphery) or eleven (core) (peri)domestic habitats had a mean bug abundance greater than 0 (domiciles, kitchens, storerooms, goat or sheep corrals, pig corrals, cow corrals, trees with chickens, chicken coops, small chicken houses, ovens, piled materials, open sheds, latrines/bathrooms, and others combined) and were included in this comparative analysis. The same type of data was collected for *T. guasayana* and *T. garciabesi* (Vazquez-Prokopec et al. 2008, Rodríguez-Planes et al. 2016). A new blanket spraying of the core and periphery with pyrethroids was conducted in April 2004, after the last data we report.

### Olta

The Olta study included detailed assessments by timed manual collections (as described above) of the relative abundance of three triatomine species (*T. infestans, T. guasayana* and *T. garciabesi*) in peridomestic structures of all rural houses over a 110 km by 75 km area eight months before and one year after spraying with different pyrethroid insecticides, diluents and spray gear (Gürtler et al. 2004). The data describe 362 inhabited houses and 15 habitats (kitchens, storerooms, chicken coops, goat or sheep corrals, pig pens, horse corrals, trees with chickens, trees without chickens, nests, mud ovens, orchards, piled materials, open sheds, latrines, and others combined), including 1,748 peridomestic sites inspected for infestation in April-May 1999 before interventions. A total of 5,251 *T. infestans* bugs were collected from 667 (38.2%) identified sites inspected at baseline. Of 352 houses which had been randomly assigned to a given treatment and sprayed with insecticide in December 1999, 325 houses were successfully re-inspected for infestation in December 2000, one year post-spraying. These houses contained 860 identified sites re-inspected for infestation after treatment, and these were included in current analyses (Table 1). Before treatment, 562 sites and 8 habitats had been infested with *T. infestans*; 139 sites and 8 habitats with *T. guasayana*, and 31 sites and 6 habitats with *T. garciabesi*.

### Figueroa

The Figueroa study included four neighboring rural communities which were assessed for infestation by timed manual collections (as described above) in October–November 2003 before a blanket spraying campaign with pyrethroid insecticides and follow-up surveys of house infestation in March and October 2004 and March 2005 (Cecere et al. 2006). The first follow-up survey included nearly all of the peridomestic sites infested pre-intervention and a fraction of the negative sites on that occasion. The following follow-up surveys additionally included domiciles partially (second survey) or completely (third survey). The data were recently reviewed for present purposes, which led to slight changes relative to the data appearing in Cecere et al. (2006). The data in Table S2 describe 201 inhabited houses from four villages and 18 habitats including 1,246 sites individually searched for triatomine bugs at baseline (Table 1). A total of 184 (14.8%) identified sites were positive for *T. infestans* and 1,274 bugs were caught at baseline. Nine habitats had mean bug abundance greater than 0 at baseline (domiciles, kitchens, storerooms, chicken coops, small chicken houses, goat corrals, pig corrals, granaries, and latrines) and were included in current analyses.

### Pampa del Indio

The following summary of the Pampa del Indio study is based on a recent complete review of the data, which led to slight changes in the numbers of habitats and infestation relative to the data published in Gurevitz et al. (2011, 2013). The Pampa del Indio study included 13 neighboring rural communities containing 353 houses (Area I) which were assessed for infestation by timed manual collections as described above in September–November 2007 before a blanket spraying campaign with pyrethroid insecticides (Gurevitz et al. 2011). The baseline data described 327 inhabited house compounds including 2,584 sites individually searched for triatomine bugs; *T. infestans* was found in 39.8% of the 327 house compounds and in 7.4% of all sites inspected, and 2,062 bugs were caught (Gurevitz et al. 2011). The preintervention survey (survey 1) combined a few data collected in a pilot survey conducted in approximately September 2007 with the massive data of October-November 2007. Table S1 includes no survey 2 for Pampa del Indio.

After initial blanket insecticide spraying, eight surveys separated by 4-5 months monitored house infestation (as described above). House compounds or sites found to be infested with *T. infestans* received selective insecticide treatments from the third survey on (August 2008) (Gurevitz et al. 2013). These surveys are summarized as surveys 3-9 in Table S1. Of 20 habitats identified, 15 had a mean bug abundance greater than 0 in at least one survey (domiciles, kitchens, storerooms, goat [and occasionally sheep] corrals, pig corrals, trees with chickens, chicken coops, small chicken houses, chicken nests, chicken roosting sticks, granaries, latrines, oven, piled materials, and others combined) and were included in current analyses (Table 1). The three-year follow-up included 20,410 identified sites (from the 20 habitats) inspected for triatomine infestation. The same type of data was collected for *T. sordida*. A unique feature of Pampa del Indio data was the detection of moderate resistance to pyrethroid insecticides in *T. infestans*. This resistance was reflected in greater house infestation than expected after initial treatment (Gurevitz et al. 2012).

### Data sets

Data for the four survey areas are included as supplementary files with summaries for *T. infestans* only of the sample means and sample variances of relative bug population density, by study, by survey, and by habitat (Table S1) and raw data for all species (Table S2). From these data, we extracted 83 sets of data from which we estimated a slope and intercept of TL (Results, Table 2), as described below. From these 83 regressions, we selected 79 with sufficient observations to support analysis of the variability of parameter estimates of individual species.

## Statistical analysis: Background on Poisson and negative binomial distributions

We use the standard statistical distinction between population and sample means, and between population and sample variances, in what follows.

If the variation in bug population size among sites of a habitat were described by a Poisson distribution with a population mean λ*_h_* that was constant for all sites of habitat *h* (e.g., for all chicken coops) but the population mean λ*_h_* differed from one habitat *h* to another (e.g., if domiciles differed from pig corrals), then, since the population mean exactly equals the population variance in the Poisson distribution, for each habitat *h* the sample mean *m_h_* should be close to the sample variance *v_h_*, i.e., *m_h_* ≈ *v_h_* ≈ λ*_h_* if samples are large enough, so log_10_ *v_h_* ≈ log_10_ *m_h_* ≈ log_10_ λ*_h_*. In this case, a plot of log_10_ *v_h_* as a function of log_10_ *m_h_* with one dot for each habitat *h* should have slope *b* not far from 1 and intercept not far from 0.

If the variation in population size among sites of each habitat were described by a negative binomial distribution with a scale parameter *k* that is constant for all habitats *h* = 1, …, *H*, and a probability parameter that changes between habitats, then the population mean μ*_h_* and the population variance σ*_h_*^2^ satisfy exactly σ*_h_*^2^ = µ*_h_* + *k*⋅μ*_h_*^2^, for *h* = 1, …, *H*. In this case, the plot of log_10_ *v_h_* as a function of log_10_ *m_h_* should have a tangent line with slope close to, but greater than, 1 for values of *m_h_* much less than 1 and a tangent line with slope close to, but less than, 2 for values of *m_h_* much greater than 1, and should curve smoothly convexly upward with increasing slope for intermediate values of *m_h_*. If the tangent line to this plot of log_10_ *v_h_* as a function of log_10_ *m_h_* has slope below 1 or above 2, it is not consistent with a negative binomial distribution of population density with constant parameter *k* and changing probability parameter unless the estimated slopes result from sampling fluctuations.

TL is not consistent with a negative binomial distribution of population density with constant parameter *k* and changing probability parameter (Cohen et al. 2016) except for population densities much smaller than 1, in which case the slope must approach 1, or for population densities much larger than 1, in which case the slope must approach 2. Analyses that assume TL can hold simultaneously with the negative binomial distribution with constant parameter *k* and changing probability parameter are internally contradictory (e.g., Wilson 1982; Wilson and Room 1983; Schultess et al. 1991).

Cohen et al. (2016) gave a proof that TL is not consistent with a negative binomial distribution of population density with a constant, positive, finite scale parameter $k=1/\rho$ and a changing probability parameter. Meng Xu (personal communication, 2017-10-19) pointed out a mistake in sign in lines numbered 25-26 of the proof. We provide a correct proof here. Cohen et al. (2016), their eq. [1]) wrote TL as

sample variance ≈ *a* × (sample mean)*^b^*, *a* > 0.

They showed correctly that the negative binomial distribution and TL are consistent if and only if $f\left( x \right)=ax^{b-2}-x^{-1}$ is constant for all $x>0$, which holds if and only if $f^{'}\left( x \right)=0$ for all $x>0$, which implies that $a=b=1$. Those values imply (from eq. (17) of Cohen et al. 2016) that $\frac{1}{\rho}=k= 0$, which excludes the negative binomial distribution except in the limiting case of a Poisson distribution, for which the population variance equals the population mean. Thus the assertion of Cohen et al. (2016) that TL is not consistent with a negative binomial distribution of population density with a constant, positive, finite scale parameter *k* is correct.

## Models: proofs of variance formulas for model 2

These proofs use repeatedly two standard formulas: *Var*(*X*) = *E*(*X*^2^) - [*E*(*X*)]^2^, for any random variable *X* that has a mean *E*(*X*) and a variance *Var*(*X*); and *E*(*XY*) = *E*(*X*)×*E*(*Y*), for any *independent* random variables *X* and *Y* that have means *E*(*X*) and *E*(*Y*) respectively.

### Proof of general variance formula after spraying for model 2

Proof that *Var*(*A*(*h*)) = *Var*(*B*(*h*))**E*([*S*(*h*)]^2^) + *Var*(*S*(*h*))*[*E*(*B*(*h*))]^2^:

*Var*(*A*(*h*)) = *Var*(*S*(*h*)**B*(*h*)) = *E*([*S*(*h*)**B*(*h*)]^2^) ‑ [*E*(*S*(*h*)**B*(*h*))]^2^

= *E*([*S*(*h*)]^2^)**E*([*B*(*h*)]^2^) ‑ [*E*(*S*(*h*)]^2^*[*E*(*B*(*h* ))]^2^

= *E*([*S*(*h*)]^2^)*{*Var*(*B*(*h*))+[*E*(*B*(*h*))]^2^} ‑ [*E*(*S*(*h*)]^2^*[*E*(*B*(*h*))]^2^

= *Var*(*B*(*h*))**E*([*S*(*h*)]^2^) + {*E*([*S*(*h*)]^2^)‑[*E*(*S*(*h*)]^2^}*[*E*(*B*(*h*))]^2^

= *Var*(*B*(*h*))**E*([*S*(*h*)]^2^) + *Var*(*S*(*h*))*[*E*(*B*(*h*))]^2^.

### Proof of variance formula after spraying for model 2 when Taylor's law holds before spraying

To prove that

*Var*(*A*(*h*)) = {*C***E*([*S*(*h*)]^2^)*[*E*(*S*(*h*))]^-^*^b^*}*[*E*(*A*(*h*))]*^b^* + {*Var*(*S*(*h*))*[*E*(*S*(*h*))]^‑2^}*[*E*(*A*(*h*))]^2^,

substitute TL, i.e.,

*Var*(*B*(*h*)) = *C**[*E*(*B*(*h*))]*^b^* for *h* = 1, 2, …, *H,*

into *Var*(*A*(*h*)) = *Var*(*B*(*h*))**E*([*S*(*h*)]^2^) + *Var*(*S*(*h*))*[*E*(*B*(*h*))]^2^ and use

*E*(*B*(*h*)) = [*E*(*S*(*h*))]^-1^**E*(*A*(*h*)).

Then

*Var*(*A*(*h*)) = *C**[*E*(*B*(*h*))]*^b^***E*([*S*(*h*)]^2^) + *Var*(*S*(*h*))*[*E*(*B*(*h*))]^2^

= *C***E*([*S*(*h*)]^2^)*[*E*(*S*(*h*))]^-^*^b^**[*E*(*A*(*h*))]*^b^* + *Var*(*S*(*h*))*{[*E*(*S*(*h*))]^-1^**E*(*A*(*h*))}^2^

= {*C***E*([*S*(*h*)]^2^)*[*E*(*S*(*h*))]^-^*^b^*}*[*E*(*A*(*h*))]*^b^* + {*Var*(*S*(*h*))* [*E*(*S*(*h*))]^-2^}**E*(*A*(*h*))^2^.

This proves the claimed formula.

If *b* = 2, then

*Var*(*A*(*h*)) = {*C***E*([*S*(*h*)]^2^)*[*E*(*S*(*h*))]^‑2^ + *Var*(*S*(*h*))*[*E*(*S*(*h*))]^‑2^}*[*E*(*A*(*h*))]^2^

= {*C***E*([*S*(*h*)]^2^) + *E*([*S*(*h*)]^2^) ‑ [*E*(*S*(*h*))]^2^}*[*E*(*S*(*h*))]^‑2^*[*E*(*A*(*h*))]^2^

= {(*C+*1)**E*([*S*(*h*)]^2^)*[*E*(*S*(*h*))]^‑2^ ‑ 1}*[*E*(*A*(*h*))]^2^.

This relation between *Var*(*A*(*h*)) and *E*(*A*(*h*)) will be an example of TL with *b* = 2 if and only if, in the above equation, the factor *E*([*S*(*h*)]^2^)*[*E*(*S*(*h*))]^‑2^ is independent of *h*, i.e., if and only if

*E*([*S*(*h*)]^2^)*[*E*(*S*(*h*))]^‑2^ = {*Var*(*S*(*h*)) + [*E*(*S*(*h*))]^2^}*[*E*(*S*(*h*))]^‑2^ = *Var*(*S*(*h*))*[*E*(*S*(*h*))]^‑2^ + 1

does not depend on *h*, and that will be true if and only if the coefficient of variation of *S*(*h*), which is the standard deviation of *S*(*h*) divided by the mean of *S*(*h*), does not depend on *h*.

# Detailed Results: Other relationships and outliers

We scanned the retained 79 numerical estimates in Table 2 for relationships and outliers among five variables: the intercept *a* of the log-log regression of TL; the slope *b* of the log-log regression of TL; the degrees of freedom (df) for each regression line (number of data points minus number of fitted parameters); the range of log_10_ mean relative abundance (the maximum log_10_ mean abundance minus the minimum log_10_ mean abundance); and the adjusted *R*^2^.

Unsurprisingly, the larger the df, the wider the range of log mean abundance and the greater the adj. *R*^2^, as shown by the positive associations in the scatterplots in Fig D, column 3, rows 4 and 5 (and conversely in the symmetrical panels in row 3, columns 4 and 5). When more kinds of habitats are available for a TL regression, it is not surprising that they have more widely varying log mean abundance and a better fit to TL. Also, the larger the df, the smaller the variability in the estimated intercepts and slopes (Fig D, column 3, rows 1 and 2; and symmetrically). More data give better agreement with TL and less variable estimates of its parameters.

The intercept *a* and the slope *b* generally increase together (Fig D, row 2, column 1, and symmetrically). This observation indicates that the higher the level of variance of relative population density (measured by the intercept *a* of TL), the greater is the proportional rate of increase in the variance for a given proportional increase in the mean (measured by the slope *b* of TL). An extreme example arose in Pampa del Indio for *T. infestans*, at the postintervention survey in October 2009 with *a* = 2.460, *b* = 2.145. This extreme example could well be due to sampling fluctuation, as *a* is not statistically significantly greater than 1 and *b* is not statistically significantly greater than 2 (Table 2).

In the lower left corner of the plot of *b* as a function of *a* (Fig D, panel in row 2, column 1), several points nearly satisfy *a* = 0, *b* = 1 (e.g., in Amamá for *T. guasayana* under pulsed control, with *a* = 0.055, *b* = 0.965; and in Figueroa, again for *T. guasayana*, postintervention in October 2004, with *a* = -0.043, *b* = 0.978). These points are consistent with a Poisson (or purely random) distribution of bugs among sites of habitat *h*, where different habitats *h* have different mean abundance λ*_h_* . As noted above, such a spatial distribution would obey a spatial TL with *a* = 0 and *b* = 1.

# SUPPLEMENTARY FIGURES

| Fig. A. For Amamá, the total (A), the mean number of *T. infestans* individuals per site of each habitat (B), and the variance of the number of *T. infestans* individuals per site of each habitat (C), in the periphery (with pulsed surveillance, vertical axis) as a function of the same summary statistics in the core (with sustained surveillance, horizontal axis), by habitat (individual dots). In all panels, the thick solid line is the diagonal line of identity between vertical and horizontal axes, *y* = *x*. Two least-squares linear regressions allow an arbitrary intercept or require the line to pass through the origin (intercept = 0). The regression equations with (y = *b*x + *a*) and without intercept (y = *b*x) are (standard error within parentheses) for (A): y = 0.7479 (0.5988)x + 62.5376 (45.6707), adj. *R*² = 0.066, and y = 1.2304 (0.5099)x, adj. *R*² = 0.349; (B): y = 1.0433 (1.1053)x + 1.1601 (0.9512), adj. *R*² = -0.014, and y = 2.0060 (0.7969)x, adj. *R*² = 0.372; (C): y = -2.2683 (3.5861)x + 92.7926 (57.4000), adj. *R*² = -0.081, and y = 1.6387 (2.9043)x, adj. *R*² = -0.082.  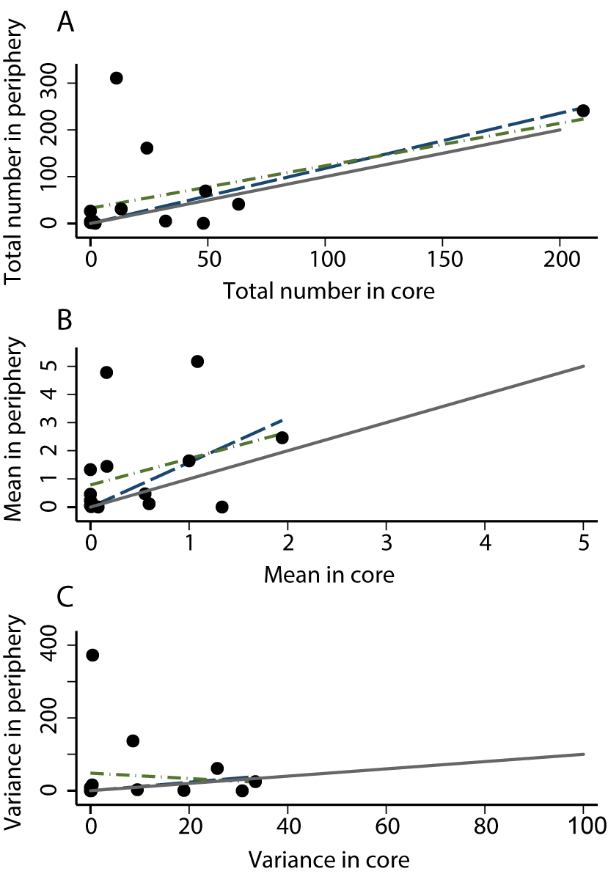 |
| --- |

| Fig. B. For Figueroa, the total (A), the mean number of *T. infestans* individuals per site of each habitat (B), and the variance of the number of *T. infestans* individuals per site of each habitat (C), before spraying (horizontal axis) and after spraying (vertical axis). The three lines are as in Fig. A. The regression equations with (y = *b*x + *a*) and without intercept (y = *b*x) are (standard error within parentheses) for (A): y = 0.0217 (0.0406)x + 11.0162 (7.2919), adj. *R*² = -0.114, and y = 0.0746 (0.0223)x, adj. *R*² = 0.559; (B): y = -0.0195 (0.0609)x + 0.2182 (0.0997), adj. *R*² = -0.147, and y = 0.0928 (0.0407)x, adj. *R*² = 0.344; (C): y = -0.0122 (0.0215)x + 2.3486 (1.0905), adj. *R*² = -0.107, and y = 0.0160 (0.0210)x, adj. *R*² = -0.055.  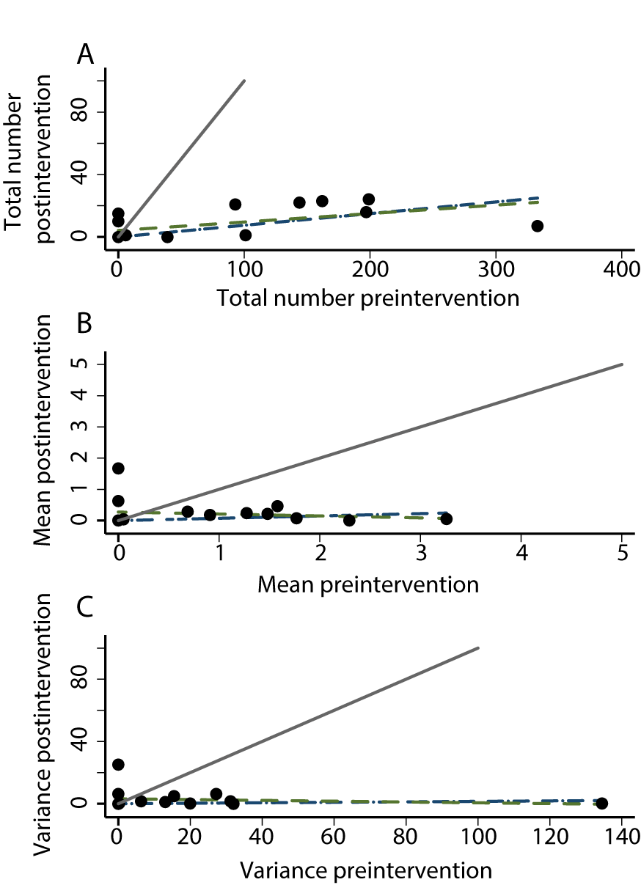 |
| --- |

| Fig. C. For Pampa del Indio, the total (A), the mean number of *T. infestans* individuals per site of each habitat (B), and the variance of the number of *T. infestans* individuals per site of each habitat (C), before spraying (horizontal axis) and after spraying (vertical axis). The three lines are as in Fig. A. The regression equations with (y = *b*x + *a*) and without intercept (y = *b*x) are (standard error within parentheses) for (A): y = 0.0377 (0.0193)x + 19.4760 (5.7378), adj. *R*² = 0.239, and y = 0.0821 (0.0208)x, adj. *R*² = 0.593; (B): y = 0.0374 (0.0495)x + 0.1468 (0.0706), adj. *R*² = -0.050, and y = 0.1255 (0.3000)x, adj. *R*² = 0.623; (C): y = 0.0387 (0.0659)x + 1.5446 (3.0925), adj. *R*² = -0.079, and y = 0.0664 (0.0340)x, adj. *R*² = 0.220.  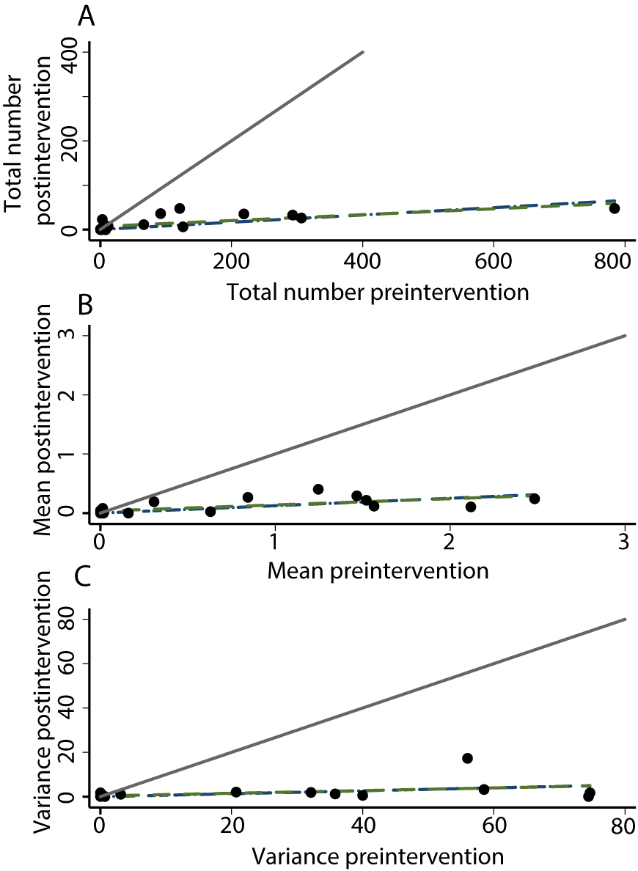 |
| --- |

| Fig. D. Scatterplot matrix and frequency histograms, for 79 single-species regressions with sufficient data, of (1) the intercept *a* of the log-log regression of TL; (2) the slope *b* of the log-log regression of TL; (3) the degrees of freedom (df) for each regression line (number of data points minus number of fitted parameters); (4) the range of log mean abundance (the maximum log mean abundance minus the minimum log mean abundance); and (5) the adjusted *R*^2^. The off-diagonal scatterplots show each variable (in the order just listed) as a function of each other variable (in the same order). For example, in the first row (counting from the top downward), second column (counting from left to right), the scatterplot shows the values of the intercept *a* (on the vertical axis) as a function of the values of the slope *b* (on the horizontal axis). The diagonal panels of Fig. D are histograms of each variable. For example, in the second row, second column, the histogram shows the frequency distribution of the slope *b*. All the histograms are unimodal, apart from minor sampling variation in counts. The histogram of adjusted *R*^2^ is left skewed, concentrated between 0.9 and 1.0, and the histogram of df is right skewed, while the other histograms are more nearly symmetrical. In these 79 regressions, the median and range of *a* are 0.962 (-0.043, 2.460) and of *b* are 1.480 (0.965, 2.145). The values of *b* outside the interval from 1 to 2 are not significantly less than 1 or greater than 2 (Table 2).  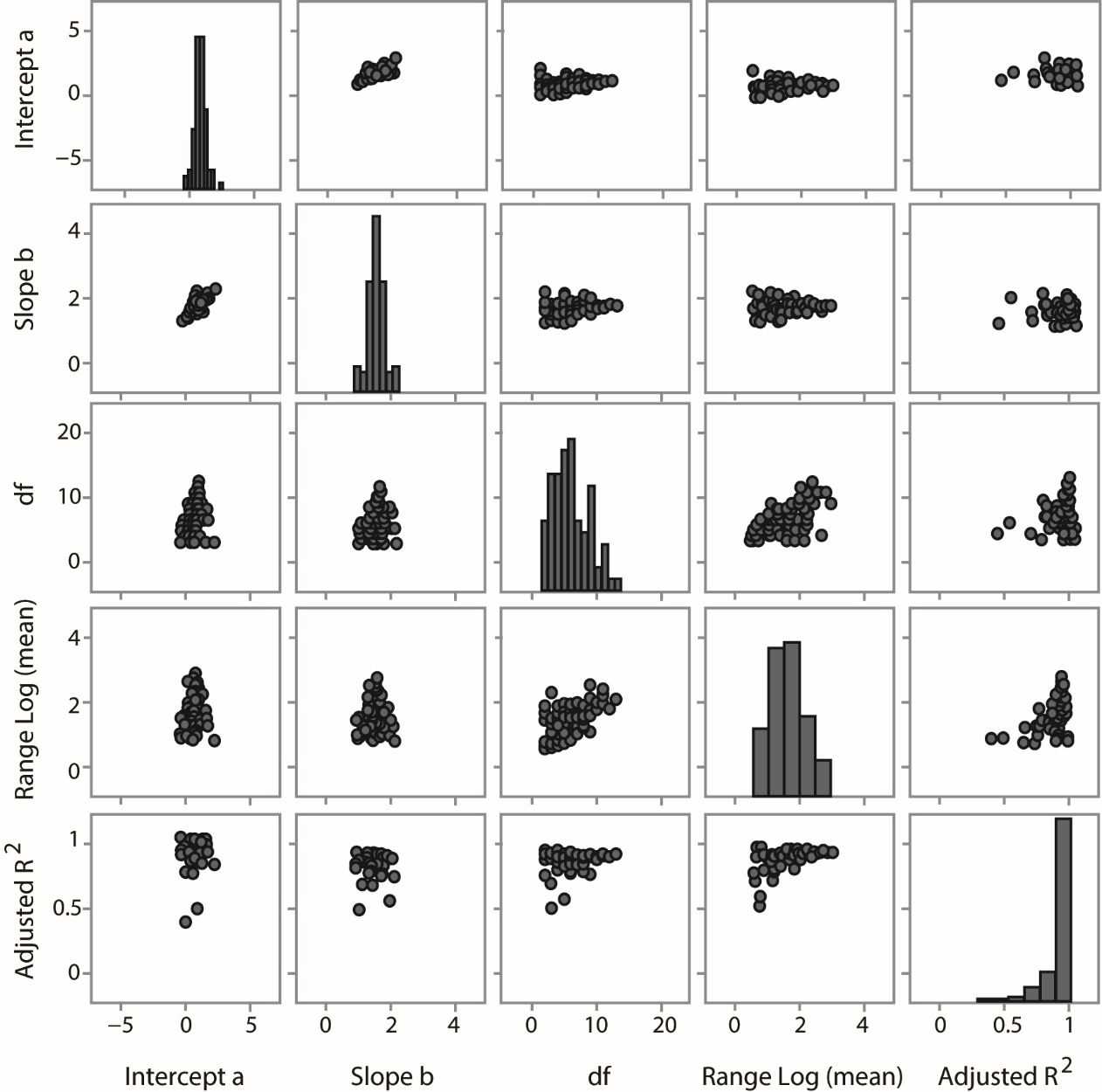 |
| --- |
